# Nusinersen mitigates neuroinflammation in severe spinal muscular atrophy patients

**DOI:** 10.1038/s43856-023-00256-2

**Published:** 2023-02-15

**Authors:** Tommaso Nuzzo, Rosita Russo, Francesco Errico, Adele D’Amico, Awet G. Tewelde, Mariangela Valletta, Amber Hassan, Michele Tosi, Chiara Panicucci, Claudio Bruno, Enrico Bertini, Angela Chambery, Livio Pellizzoni, Alessandro Usiello

**Affiliations:** 1 Department of Environmental, Biological and Pharmaceutical Sciences and Technologies, University of Campania “Luigi Vanvitelli”, Caserta, Italy; 2 Laboratory of Translational Neuroscience, Ceinge Biotecnologie Avanzate Franco Salvatore, Naples, Italy; 3 Department of Agricultural Sciences, University of Naples “Federico II”, Portici, Italy; 4 Unit of Neuromuscular and Neurodegenerative Disorders, Dept. Neurosciences, Bambino Gesu’ Children’s Hospital IRCCS, Roma, Italy; 5 Center of Translational and Experimental Myology, IRCCS Istituto Giannina Gaslini, Genoa, Italy; 6 Department of Neuroscience, Rehabilitation, Ophthalmology, Genetics, Maternal, and Child Health - DINOGMI, University of Genoa, Genoa, Italy; 7 Center for Motor Neuron Biology and Disease, Columbia University, New York, NY USA; 8 Department of Pathology and Cell Biology, Columbia University, New York, NY USA; 9 Department of Neurology, Columbia University, New York, NY USA

**Keywords:** Motor neuron disease, Cytokines

## Abstract

**Background:**

Neuroinflammation contributes to the onset and progression of neurodegenerative diseases, but has not been specifically investigated in patients affected by severe and milder forms of spinal muscular atrophy (SMA).

**Methods:**

In this two-center retrospective study, we investigated signatures of neuroinflammation in forty-eight pediatric male and female SMA1 (*n* = 18), male and female SMA2 (*n* = 19), and female SMA3 (*n* = 11) patients, as well as in a limited number of male and female non-neurological control subjects (*n* = 4). We employed a Bio-Plex multiplex system based on xMAP technology and performed targeted quantitative analysis of a wide range of pro- and anti-inflammatory cytokines (chemokines, interferons, interleukins, lymphokines and tumor necrosis factors) and neurotrophic factors in the cerebrospinal fluid (CSF) of the study cohort before and after Nusinersen treatment at loading and maintenance stages.

**Results:**

We find a significant increase in the levels of several pro-inflammatory cytokines (IL-6, IFN-γ, TNF-α, IL-2, IL-8, IL-12, IL-17, MIP-1α, MCP-1, and Eotaxin) and neurotrophic factors (PDGF-BB and VEGF) in the CSF of SMA1 patients relative to SMA2 and SMA3 individuals, who display levels in the range of controls. We also find that treatment with Nusinersen significantly reduces the CSF levels of some but not all of these neuroinflammatory molecules in SMA1 patients. Conversely, Nusinersen increases the CSF levels of proinflammatory G-CSF, IL-8, MCP-1, MIP-1α, and MIP-1β in SMA2 patients and decreases those of anti-inflammatory IL-1ra in SMA3 patients.

**Conclusions:**

These findings highlight signatures of neuroinflammation that are specifically associated with severe SMA and the neuro-immunomodulatory effects of Nusinersen therapy.

## Plain Language Summary

Spinal muscular atrophy (SMA) is an inherited disorder which leads to muscle weakening. Three therapies have recently been developed, including Nusinersen. However, the effect of SMA on the immune system and how this could be affected by Nusinersen is unknown. The immune system protects the body from infection and, in some disorders, misfunctions and damages the body in the absence of infection. Here, we analyze components of the immune system in body fluids from SMA patients before and after treatment with Nusinersen. The immune system was found to be more active in patients with more severe disease. Treatment with Nusinersen reduced the levels of some, but not all of these, components of the immune system. Thus, treatments that impact the immune system might improve symptoms in patients with SMA.

## Abstract (web summary)

Nuzzo, Russo, Errico, D’Amico et al. investigate neuroinflammation in forty-eight pediatric spinal muscular atrophy patients before and after Nusinersen treatment. They find signatures of neuroinflammation that are specifically associated with severe disease and show that Nusinersen therapy has neuro-immunomodulatory effects.

## Introduction

Spinal Muscular Аtrорhy (SMА) is an autosomal recessive neurodegenerative disorder caused by homozygous deletions or mutations in the *survival motor neuron 1* (*SMN1*) gene^1^. All SMA patients lack a functional *SMN1* gene and retain one or more copies of the *SMN2* gene, which produces insufficient amounts of the SMN protein leading to motor neuron loss, skeletal muscle weakness, аtrорhy and multiorgan abnormalities^2,3^. SMA patients display a wide range of clinical manifestations and are classified into three main groups (types 1, 2 and 3) according to the age of onset and maximum motor function achieved^2–4^. Disease severity inversely correlates with the levels of *SMN* expression and the number of copies of *SMN2*^2,3,5^.

Current SMA therарies increase expression of SMN protein through multiple mechanisms and induce significant clinical improvement in SMA patients treated pre-symptomatically, while delayed treatment often results in a more variable clinical response^6,7^. However, the metabolic effects of these drug treatments are poorly defined and may shed light on strengths and limitations governing therapeutic efficacy as well as disease mechanisms.

Nusinersen—the first FDA-approved drug for the treatment of SMА—is an antisense oligonucleotide that increases SMN expression through modulation of *SMN2* pre-mRNA splicing and is administered intrathecally at regular intervals after an initial loading phase^8–10^. As withdrawal of cerebrospinal fluid (CSF) is routinely performed at the time of each scheduled injection, the collected samples allow investigating the biochemical composition of this biofluid in naive patients prior to treatment as well as longitudinal changes induced by the therapy.

In this study, we sought to investigate signatures of neuroinflammation as a clinically relevant yet understudied aspect of SMA pathology in relation to disease severity and Nusinersen treatment. Accordingly, while neuroinflammation has been implicated in the onset and progression of neurodegenerative diseases such as multiple scleroris (MS) and amyotrophic lateral sclerosis (ALS) as well as Alzheimer’s (AD) and Parkinson’s (PD)^11–13^, it has not been thoroughly investigated in pediatric SMA patients^14–16^.

To address this outstanding issue, we profiled a wide range of pro- and anti-inflammatory cytokines (chemokines, interferons, interleukins, lymphokines and tumor necrosis factors) and neurotrophic factors in the CSF of SMA patients across the spectrum of clinical severity before and after treatment with Nusinersen. Our analysis documents neuroinflammatory signatures specifically associated with severe SMA and distinct neuro-immunomodulatory effects of Nusinersen therapy in patients of different clinical severity.

## Methods

### Patients′ characteristics

This two-center study (Bambino Gesù Hospital, Rome, Italy; Giannina Gaslini Institute, Genoa, Italy) was carried out on forty-eight SMA1 (*n* = 18), SMA2 (*n* = 19) and SMA3 (*n* = 11) patients receiving intrathecal administration of Nusinersen (12 mg) (Table 1). The study was approved by the local Ethics Committees of the two Hospitals (2395_OPBG_2021). CSF samples were collected on day 0 (T0; first Nusinersen injection, baseline), day 64 (T1; fourth Nusinersen injection) and day 302 (T2; sixth Nusinersen injections) and assessed for cytokines detection. All patients underwent clinical diagnosis that was confirmed through genetic analysis of *SMN2* copy number. All SMA1 patients were involved in the Expanded Access Programme (EAP) for compassionate use to patients with the infantile form of the disease. EAP was carried out in Italy between November 2016 and November 2017. Clinical response to Nusinersen of SMA1, SMA2 and SMA3 patients involved in this study has been previously described^17–19^. In addition, non-neurological pediatric control subjects ranging in age from 2–12 years (*n* = 4), were used as a reference for physiological baseline levels of cytokines but were not included in statistical analyses due to their low number (Table 1). All participants and/or their legal guardians signed a written informed consent. We found significant differences in age among all SMA types (SMA1 vs SMA2, *p* = 0.015; SMA1 vs SMA3, *p* < 0.0001; SMA2 vs SMA3, *p* = 0.013; Mann–Whitney test) and in the body mass index (BMI) of SMA1 patients compared to SMA2 or SMA3 patients (*p* = 0.002 and *p* = 0.003, respectively; Mann–Whitney test). Differences in sex (*χ*^2^ = 5.895, *p* = 0.052), SMN2 copy number (*χ*^2^ = 36.602, *p* < 0.0001), and proportion of patients who underwent gastrostomy (*χ*^2^ = 32.941, *p* < 0.0001) or tracheostomy (*χ*^2^ = 16.000, *p* < 0.0001) were also significant among SMA groups. In contrast, no effect of non-invasive ventilation (NIV) was found (*χ*^2^ = 2.839, *p* = 0.242).

**Table 1:** Demographic and clinical characteristics of naive SMA subjects (total *n* = 48) enrolled in the study.

	Control (*n* = 4)	SMA1 (*n* = 18)	SMA2 (*n* = 19)	SMA3 (*n* = 11)
	Median [Min; Max)	Median [Min; Max)	Median [Min; Max)	Median [Min; Max)
Sex (*n*)	3F/1M	11F/7M	12F/7M	11F/0M
Age (Years)	7.5 [2.5;12]	3.0 [0.1;7.7]	5.4 [0.9;13.3]	12.6 [2.2;17.9]
BMI		13.5 [11.5;18.5]	17.5 [10.6;28.9] (*n* = 17)	19.7 [10.7;26.5] (*n* = 10)
*SMN2* copy (2/3/4)	–	17/1/0	2/17/0	2/7/2
CHOP-INTEND	–	7.5 [0;52]	–	–
HFMSE	–	–	9 [2;41]	45 [15;62]
Gastrostomy (*n*)	–	4 No/14 Yes	19 No/0 Yes	11 No/0 Yes
NIV (*n*)	–	12 No/6 Yes	12 No/7 Yes	10 No/1 Yes
Tracheostomy (*n*)	–	10 No/8 Yes	19 No/0 Yes	11 No/0 Yes

Values are expressed as median (min; max). For sex, gastrostomy, tracheostomy and NIV, number of subjects (*n*) is indicated. Statistical analyses were performed by Chi-square test or Mann–Whitney test.

*BMI* body max index, *CHOP-INTEND* Children’s Hospital Of Philadelphia Infant Test Of Neuromuscular Disorders, *HFMSE* Hammersmith Functional Motor Scale Expanded, *NIV* non-invasive ventilation.

### Clinical evaluation

Clinical assessment of patients, including anthropometric measurements and vital parameters, was performed at T0, T1, and T2 by experienced child neurologists or pediatricians expert of SMA, which also recorded feeding (oral nutrition, nasogastric tube or percutaneous gastrostomy) and nutritional status (BMI), as well as respiratory function (spontaneous breathing, NIV or tracheostomy). Of the eighteen SMA1 patients, one was 1 month-old, while the others were from 6 months to 7 years and 8 months old at baseline. Eight patients were subjected to tracheostomy and 6 to NIV (<16 h/day). Fourteen patients had gastrostomy, and all were underweight (BMI < 18.5). The nineteen SMA2 patients ranged from 11 months to 13.3 years at the beginning of treatment. Twelve patients were in spontaneous breathing while seven were subjected to NIV. None of them had tracheostomy or gastrostomy. Eleven patients had a BMI below 18. Of the eleven SMA3 patients, one was non ambulant and one was under NIV (<16 h/day). None had gastrostomy. Functional motor assessments, chosen according to age and motor function of patients, were performed at T0, T1, and T2 by expert physiotherapists trained with standardized procedure manual^20^ and reliability sessions. SMA1 patients were assessed through the Children’s Hospital of Philadelphia Infant Test of Neuromuscular Disorders (CHOP-INTEND)^21,22^, a functional scale that includes 16 items assessing motor function in weak infants. The score of each item is between 0 and 4 (0: no response, 4: complete level of response), with a total score ranging from 0 to 64. Motor assessment of SMA2 and SMA3 patients was evaluated by the Hammersmith Functional Motor Scale Expanded (HFMSE)^22,23^, including 33 items that investigate the ability to perform different activities. The total score ranges from zero (all activities are failed) to 66 (better motor function). During motor evaluations, all patients did not wear spinal jackets or orthoses.

### Intrathecal Nusinersen treatment

Nusinersen (12 mg/dose) was administered under hospital environment. Fasting preceding the lumbar puncture was <4 h in SMA1 patients and was planned before the procedure, while it was 6–8 h in SMA2 and SMA3 patients. In SMA1 the intrathecal injection was carried without sedation, while in SMA2 and SMA3 patients an intravenous midazolam sedation was applied in a mean interval of 3–5 min, during which the CSF sampling was performed. Patients did not experience severe adverse effects. To avoid post-lumbar puncture symptoms, all patients laid down for 2 h after the infusion.

### CSF sample collection and sample characteristics

CSF sample collection was carried out at the time of intrathecal Nusinersen treatment in polypropylene tubes and stored at −80 °C before being further analyzed. Detection of pH, total protein content, and cytokines and chemokines levels was performed on the CSF sample of each patient. Determination of total protein levels was carried out by Bradford assay (Bio-Rad, München, Germany)^24^. CSF cell count was not available for this retrospective study. The CSF samples from all groups did not differ in pH (SMA1 vs SMA2, *p* = 0.475; SMA1 vs SMA3, *p* = 0.893; SMA2 vs SMA3, *p* = 0.401; Mann–Whitney test) and total protein content (total proteins: SMA1 vs SMA2, *p* = 0.670; SMA1 vs SMA3, *p* = 0.938; SMA2 vs SMA3, *p* = 0.863; Mann–Whitney test) (Supplementary Table 1). Of the eighteen SMA1 patients, we collected *n* = 18 CSF samples at T0, *n* = 15 at T1, and *n* = 16 at T2. Of the nineteen SMA2 patients, we collected *n* = 19 CSF samples at T0, *n* = 11 at T1, and *n* = 18 at T2. Of the eleven SMA3 patients, we collected all CSF samples at T0, T1, and T2 (Supplementary Table 1). Exclusion criteria included the presence of symptoms or changes in blood biochemical and hematological parameters suggestive of a systemic inflammatory state, and/or immunosuppressive treatments ongoing in the last 6 months before inclusion.

### Targeted quantitative analysis of secreted cytokines by Bio-Plex assay

The targeted quantitative analysis of secreted cytokines and chemokines in CSF samples was performed using the Bio-Plex multiplex system (Bio-Rad, Milan, Italy) based on xMAP technology^25^. Magnetic beads labeled with red and infrared fluorophores, are coated with specific antibodies, thus allowing the simultaneous detection of multiple target analytes within one sample. Following reaction of beads with target analytes, detection is performed with a biotinylated antibody and phycoerythrin conjugated streptavidin. All steps were performed according to manufacturer’s instructions. The concentration of the following analytes were detected simultaneously: interleukin (IL)−1β, IL-1ra, IL-2, IL-4, IL-5, IL-6, IL-7, IL-8, IL-9, IL-10, IL-12 (p70), IL-13, IL-15, IL-17A, IP10, eotaxin, granulocyte-colony stimulating factor (G-CSF), Granulocyte macrophage colony stimulating factor (GM-CSF), Interferon (IFN)γ, monocyte chemoattractant protein 1 (MCAF/MCP-1), Macrophage inflammatory protein 1-alpha and beta (MIP-1α and MIP-1β), RANTES, tumor necrosis factor alpha (TNF-α), platelet-derived growth factor-BB (PDGF-BB), Vascular endothelial growth factor (VEGF), Basic fibroblast growth factor (FGF-basic). Each sample was tested in triplicate, and each plate contained a balanced sample size for SMA type and treatment groups. Data were acquired using a Bio-Plex MAGPIX Multiplex Reader system (Bio-Rad). Standard curves optimization and the calculation of analyte concentrations were performed by using the Bio-Plex Manager software. Cytokine concentration was expressed in pg/ml.

### Statistics and reproducibility

The normality distribution was tested using the Kolmogorov–Smirnov test. Continuous variables were shown as median (interquartile range, IQR). In untreated SMA groups, comparisons in the levels of cytokines and neurotrophic factors among SMA1, SMA2 or SMA3 patients were evaluated by ANCOVA considering age, sex, BMI, *SMN2* copies number, gastrostomy and tracheostomy/NIV as covariates. Cytokines or neurotrophic factors showing significant *p*-values (*p* < 0.05) from ANCOVA analysis, were then analyzed by Bonferroni-corrected pairwise comparisons. Differences in continuous variables between Nusinersen treatment or within SMA groups were evaluated by non-parametric Wilcoxon matched-pairs signed ranks test or Mann–Whitney test. Spearman’s non-parametric correlation was used to test possible associations between continues variables. Benjamini-Hochberg procedure was used to decrease the false discovery rate and avoid Type I errors (false positives).

### Reporting summary

Further information on research design is available in the Nature Portfolio Reporting Summary linked to this article.

## Results

### Levels of cytokines and neurotrophic factors in the CSF of naive SMA patients

**Fig. 1:**
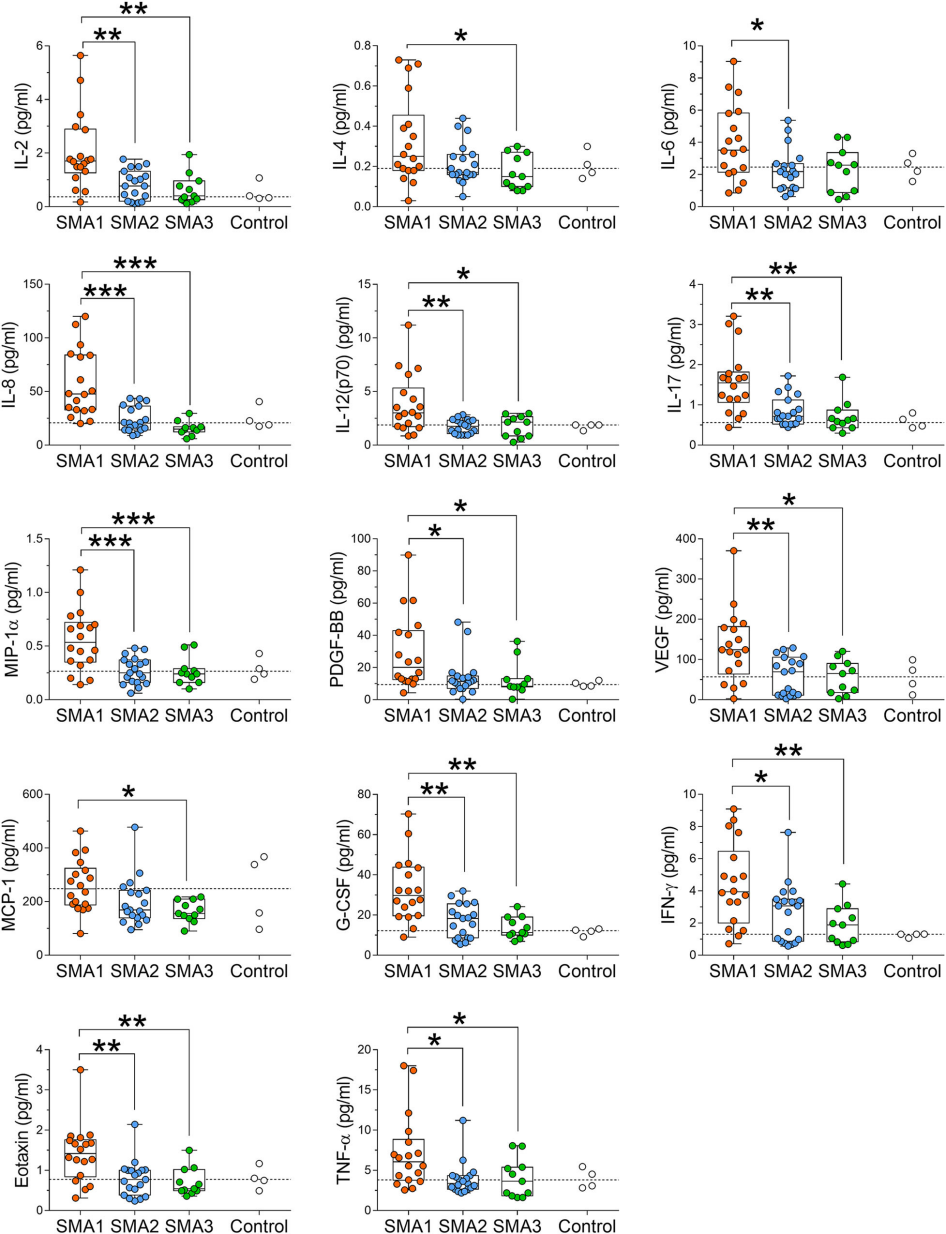
Levels of cytokines and neurotrophic factors in the CSF of naive SMA patients. Increased levels of IL-2, IL-4, IL-6, IL-8, IL-12 (p70), IL-17, MIP-1α, PDGF-BB, VEGF, MCP-1, G-CSF, IFN-γ, eotaxin and TNF-α are found in the CSF of SMA1 (*n* = 18) patients compared to SMA2 (*n* = 19) and/or SMA3 (*n* = 11) patients. Controls (*n* = 4) were used as a reference for physiological levels of cytokines. Data are shown as box and whisker plots representing median with interquartile range (IQR). *p < 0.05, **p < 0.01; ***p < 0.0001, compared to SMA2 or SMA3 (Bonferroni multiple comparisons). Dots represent individual patients’ values. The dotted line represents the median of control samples.

We performed targeted quantitative analysis of several neuroinflammatory markers in CSF samples from SMA1 (*n* = 18), SMA2 (*n* = 19) and SMA3 (*n* = 11) patients (Table 1) using a Bio-Plex multiplex system based on xMAP technology^25^. CSF samples from non-neurological pediatric control subjects (*n* = 4) were analyzed as a reference for physiological baseline levels of cytokines but were not included in statistical analyses due to their low number. Considering age, sex, BMI, *SMN2* copy number, gastrostomy and tracheostomy/NIV as covariates, multivariate adjusted ANCOVA analysis showed significant differences among SMA groups in the basal CSF levels of IL-2, IL-4, IL-6, IL-8, IL-12, IL-17, MIP-1α, PDGF-BB, VEGF, MCP-1, G-CSF, IFN-γ, Eotaxin, TNF-α, and IP-10 (Supplementary Table 2). Following Bonferroni multiple comparisons, we found increased levels of pro-inflammatory molecules IL-2, IL-6, IL-8, IL-12, IL-17, MIP-1α, PDGF-BB, VEGF, G-CSF, IFN-γ, eotaxin, and TNF-α in the CSF of SMA1 patients compared to SMA2 (Fig. 1; Supplementary Table 2). Similarly, the levels of IL-2, IL-4, IL-8, IL-12, IL-17, MIP-1α, PDGF-BB, VEGF, MCP-1, G-CSF, IFN-γ, eotaxin, and TNF-α were increased in the CSF of SMA1 patients compared to SMA3 (Fig. 1; Supplementary Table 2). Conversely, no significant differences were found between SMA2 and SMA3 patients for all cytokines analyzed, whose concentration values were comparable with those of healthy pediatric individuals (Fig. 1; Supplementary Fig. 1; Supplementary Table 2).

Next, we evaluated if the observed cytokine variations are associated with demographic and clinical features of patients, including age, BMI, tracheostomy/NIV, and gastrostomy. Mann–Whitney analysis showed that the levels of cytokines were similar in severe SMA patients with or without tracheostomy/NIV or gastrostomy (Supplementary Table 3). Spearman’s correlations analysis showed a positive correlation of MCP-1, MIP-1β and RANTES levels with the BMI of SMA1 patients after correction for Benjamini-Hochberg (B-H) multiple comparisons (MCP-1: *r* = 0.674, *p* = 0.002, B-H, *p* = 0.006; MIP-1β: *r* = 0.717, *p* = 0.001, B-H, *p* = 0.002; RANTES: *r* = 0.680, *p* = 0.002, B-H *p* = 0.004) selectively at T0 (Supplementary Table 4). Conversely, no correlations were found between cytokines levels and BMI or age of SMA patients evaluated by Spearman’s correlations after correction for Benjamini-Hochberg (B-H) multiple comparisons (Supplementary Tables 4–6). No correlations were also observed between the levels of cytokines or neurotrophic factors and basal motor function assessed with CHOP-INTEND in SMA1 patients (Supplementary Table 4), and HFMSE in SMA2 and SMA3 patients (Supplementary Tables 5 and 6).

Collectively, these results identify upregulation of a set of potent pro-inflammatory signaling molecules that is specific to SMA1 patients when compared to later onset, milder SMA patients, pointing to neuroinflammation as a specific feature of the most severe form of the disease.

### Effect of Nusinersen treatment on the levels of cytokines and neurotrophic factors in the CSF of SMA patients

**Fig. 2:**
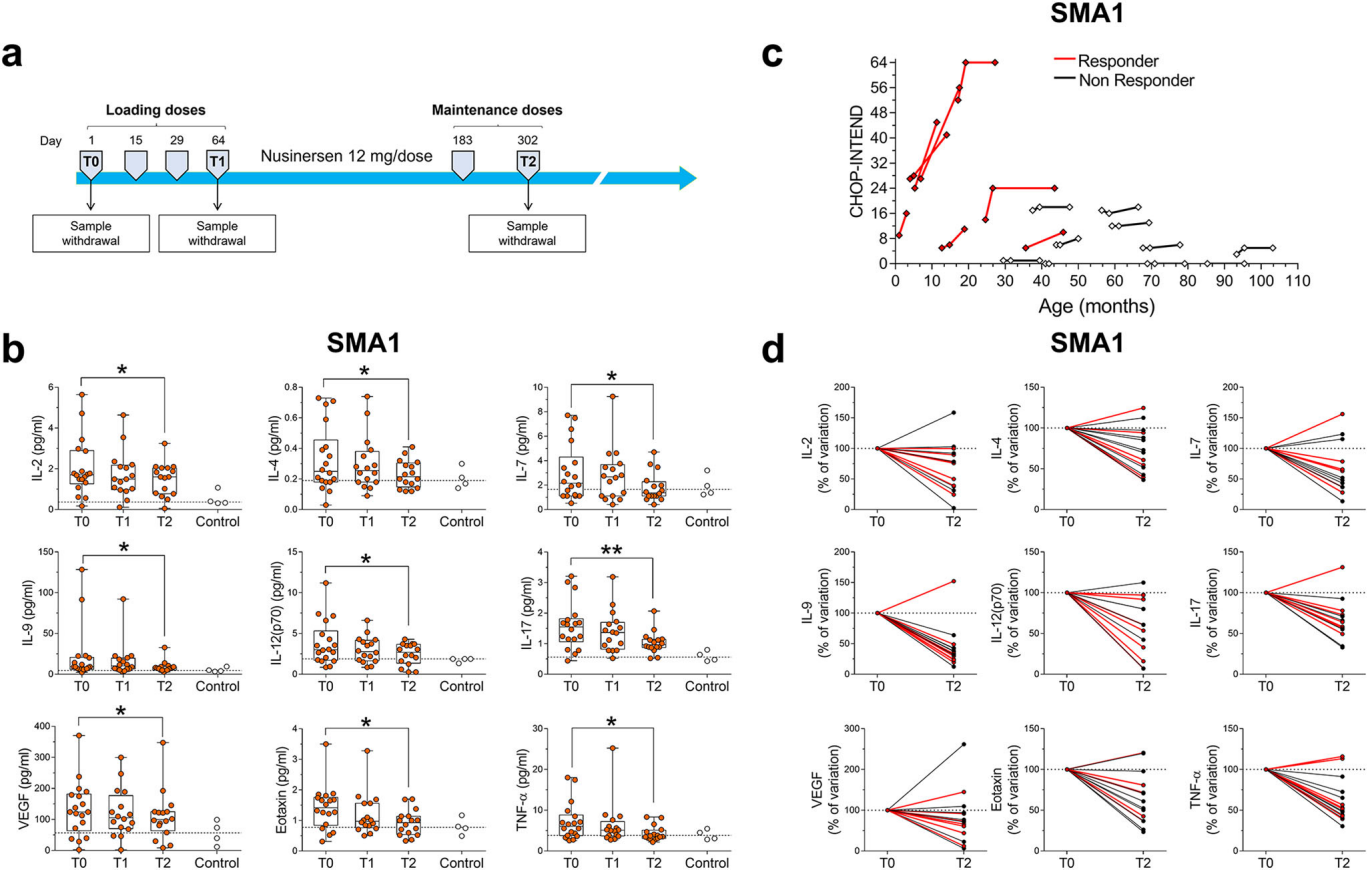
Nusinersen decreases the levels of a subset of cytokines and neurotrophic factors in the CSF of SMA1 patients. **a** Schematic representation of the timeline of intrathecal Nusinersen administration and CSF collection in SMA patients. **b** Levels of IL-2, IL-4, IL-7, IL-9, IL-12, IL-17, VEGF, eotaxin, and TNF-α in the CSF of SMA1 patients prior to treatment (T0, *n* = 18) and at the time of the fourth (T1, *n* = 16) and the sixth (T2, *n* = 16) injection of Nusinersen. Controls (*n* = 4) were used as a reference for physiological levels of cytokines. *p < 0.05, **p < 0.01, com*p*ared to T0 (Wilcoxon matched-pairs signed ranks test). Data are shown as box and whisker plots representing median with interquartile range (IQR). Dots represent individual patients’ values. The dotted line represents the median of control samples. **c** Spaghetti plots representing the CHOP-INTEND score of SMA1 patient at T0, T1, and T2. **d** Spaghetti plots showing variations of cytokines levels in the CSF of SMA1 patients between T0 and T2. Cytokines variations are expressed as percentage of the T0 values (expressed as 100%). Patients are indicated as “responders” (red lines) or “non-responders” (black lines) to Nusinersen therapy.

We next performed a longitudinal analysis of the effects of Nusinersen treatment on the CSF levels of cytokines and neurotrophic factors in the CSF of SMA patients. To do so, we analyzed CSF samples collected 64 (T1) and 302 (T2) days after the first injection was performed (T0), corresponding to loading and maintenance phases of the drug administration protocol, respectively (Fig. 2a). Remarkably, according to the Wilcoxon matched-pairs signed ranks test, we found a significant decrease in the CSF levels of different inflammatory molecules such as IL-2, IL-4, IL-7, IL-9, IL-12, IL-17, VEGF, eotaxin and TNF-α, in Nusinersen-treated SMA1 patients at T2 relative to their baseline concentrations, (Fig. 2b; Supplementary Table 7). Furthermore, there was no significant effect of age, BMI, tracheostomy/NIV or gastrostomy on these changes as analyzed by linear regression or Mann–Whitney analysis (Supplementary Tables 3 and 4). To investigate whether the reduction in the pro-inflammatory secreted molecules induced by Nusinersen correlated with clinical outcomes, we first stratified SMA1 patients into “responders” and “non-responders” to therapy—with responders having an increase of at least 4 points in the CHOP-INTEND scale at T2 relative to T0 (Fig. 2c). We then analyzed the percent variation in the levels of cytokines between T0 and T2 in each patient and found that most Nusinersen-treated SMA1 patients showed a reduction in the CSF levels of pro-inflammatory molecules regardless of their improvement in CHOP-INTEND scores (Fig. 2d). Consistent with the lack of association between cytokine levels and clinical response to Nusinersen, Mann–Whitney analysis revealed comparable cytokines and neurotrophic factors levels between SMA1 patients responsive or not responsive to Nusinersen (Supplementary Table 8). Together, these results reveal that Nusinersen mitigates neuroinflammation in the CSF of SMA1 patients. However, the increased basal levels of a subset of pro-inflammatory cytokines such as IL-6, IL-8, G-CSF, IFNγ, MCP1, MIP-1α, and the neurotrophic factor PDGF-BB are not affected by Nusinersen (Supplementary Fig. 2; Supplementary Table 7), suggesting a potential shortcoming of this therapy.

**Fig. 3:**
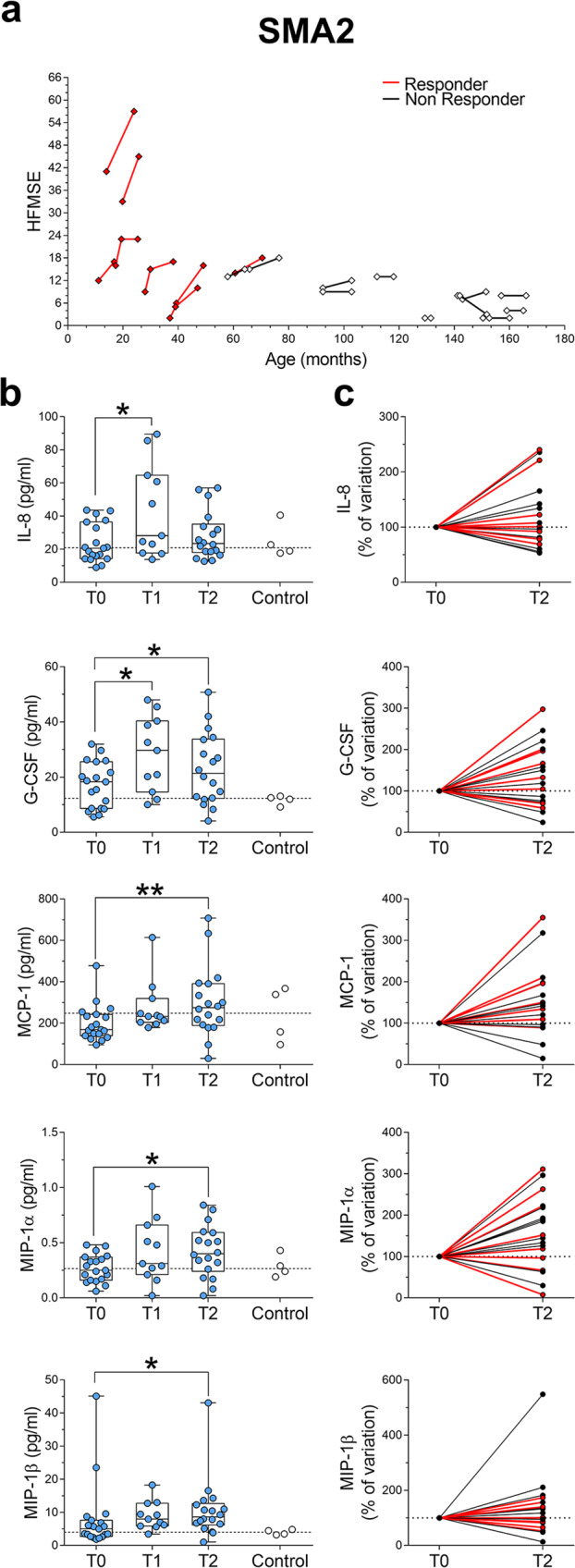
Nusinersen treatment increases the levels of several pro-inflammatory cytokines in the CSF of SMA2 patients. **a** Spaghetti plots representing the HFMSE score of each SMA2 patient at T0 (*n* = 19) and at the fourth (T1, *n* = 11) and/or sixth (T2, *n* = 18) injection of Nusinersen. **b** Nusinersen increases the levels of IL-8, G-CSF, MCP-1, MIP-1α, and MIP-1β in the CSF of SMA1 patients at T1 and/or T2 relative to T0. Controls (*n* = 4) were used as a reference for physiological baseline levels of cytokines. The dotted line represents the median of control samples. Data are shown as box and whisker plots representing median with interquartile range (IQR). Dots represent individual patients’ values. *p < 0.05, **p < 0.01, com*p*ared to T0 (Wilcoxon matched-pairs signed ranks test). **c** Spaghetti plots showing variations of cytokines levels in the CSF of SMA2 patients between T0 and T2. Cytokines variations are expressed as percentage of the T0 values (expressed as 100%). Patients were indicated as “responders” (red lines) or “non-responders” (black lines) to Nusinersen therapy.

**Fig. 4:**
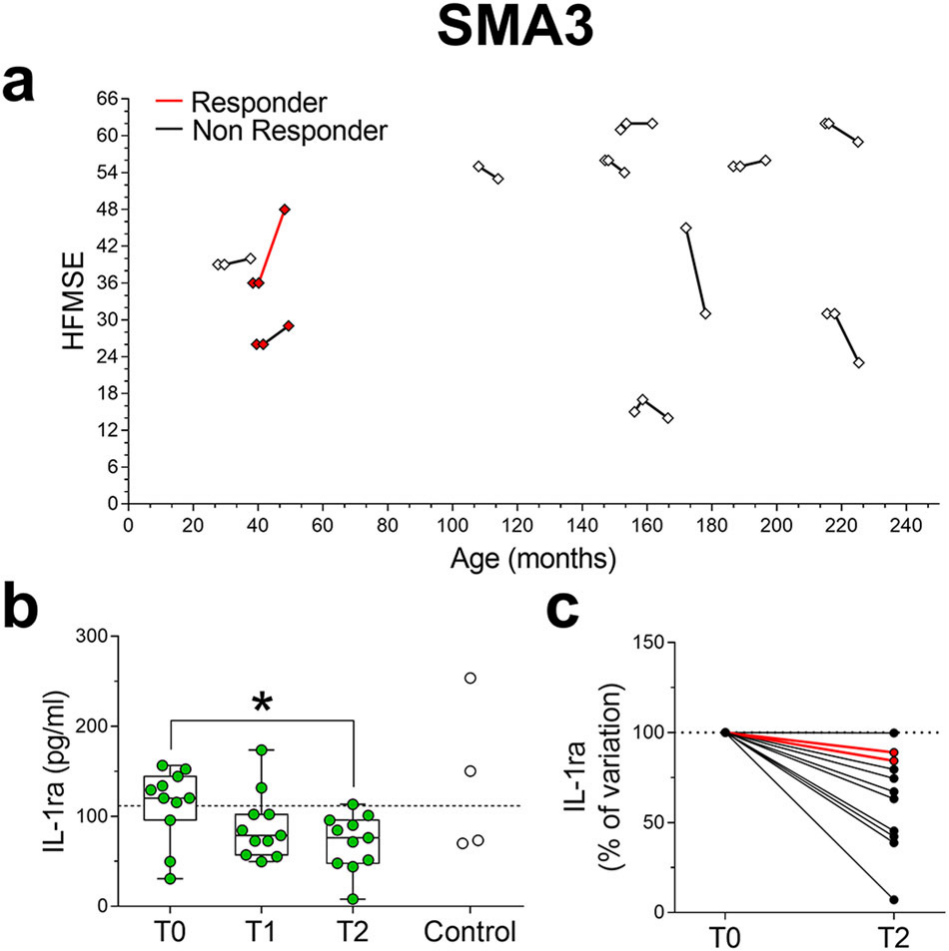
IL-1ra levels are decreased by Nusinersen treatment in the CSF of SMA3 patients. **a** Spaghetti plots representing the HFMSE score of each SMA3 patient prior to treatment (T0, *n* = 11) at the fourth (T1, *n* = 11) and/or sixth (T2, *n* = 11) injection of Nusinersen. **b** Levels of IL-1ra in the CSF of SMA3 patients prior to treatment (T0) and at the time of the fourth (T1) and the sixth (T2) injection of Nusinersen. Controls (*n* = 4) were used as a reference for physiological baseline levels of cytokines. The dotted line represents the median of control samples. Data are shown as box and whisker plots representing median with interquartile range (IQR). Dots represent individual patients’ values. *p < 0.05, compared to T0 (Wilcoxon matched-pairs signed ranks test). **c** Spaghetti plots showing variations of IL-1ra levels in the CSF of SMA3 patients between T0 and T2. Cytokines variations are expressed as percentage of the T0 values (expressed as 100%). Patients were indicated as “responders” (red lines) or “non-responders” (black lines) to Nusinersen therapy.

We next analyzed the effects of Nusinersen on the profile of inflammatory molecules in the CSF of milder SMA patients. In SMA2 patients, Wilcoxon matched-pairs signed ranks test showed a significant increase in the CSF levels of IL-8, G-CSF, MCP-1, MIP-1α, and MIP-1β levels after Nusinersen treatment relative to their concentrations prior to therapy (Fig. 3; Supplementary Fig. 3; Supplementary Table 7). In SMA3 patients, Nusinersen had a specific effect on the anti-inflammatory IL-1ra, the levels of which were reduced at T2 (Fig. 4; Supplementary Fig. 4; Supplementary Table 7). We did not find any correlation between changes in cytokines levels and either BMI or age in Nusinersen-treated SMA2 and SMA3 patients (Supplementary Tables 5 and 6). Similarly, Mann–Whitney analysis showed that the levels of cytokines were similar in Nusinersen-treated SMA2 patients with or without NIV (Supplementary Table 3). Moreover, the Nusinersen-induced effects on cytokine levels did not correlate with differences in clinical response to treatment defined by an increase of at least 3 points in the HFMSE scale at T2 relative to baseline in both SMA2 (Fig. 3; Supplementary Table 8) and SMA3 patients (Fig. 4; Supplementary Table 8). Thus, while there are no significant differences in the levels of pro-inflammatory and anti-inflammatory cytokines or neurotrophic factors between SMA2 and SMA3 patients prior to treatment, Nusinersen appears to paradoxically trigger distinct immunological responses in milder SMA patients, which may reflect off target effects of this drug in the CNS.

## Discussion

Neuroinflammation represents a critical factor in the onset and progression of neurodegenerative diseases^26^. However, despite being the most common neurodegenerative pediatric disorder^3^, there has been a lack of knowledge of possible neuroinflammatory components in SMA. To fill this gap, here we evaluated the CSF levels of a wide range of pro-inflammatory and anti-inflammatory interleukins, chemokines, and growth factors in a cohort of SMA patients from two Italian Hospitals stratified according to clinical severity. Our study reveals a remarkable increase in the CSF levels of potent pro-inflammatory cytokines that is specific to SMA1 patients relative to individuals affected by milder forms of the disease. These findings highlight an immunological disease feature of severe SMA, which links the magnitude of SMN protein deficiency to neuroinflammation. Consistent with a potential contribution to SMA pathogenesis, increased levels of the same neuroinflammatory cytokines have been documented in the CSF of patients with other neurodegenerative diseases^11–13^. Furthermore, since increased levels of IL-2, IL-8, IL-17, G-CSF, MCP-1, MIP-1α, and VEGF were previously identified in the CSF of ALS patients^13^, these molecules emerge as shared neuroinflammatory signatures across motor neuron diseases^14–16^.

Our findings suggest widespread dysregulation of cytokines levels in the CNS of severe SMA patients rather than a restricted effect on select secreted molecules of the neuroinflammatory response^27^. Accordingly, we found that several pro-inflammatory interleukins, chemokines and growth factors (IL-2, IL-6, IL-8, IL-12, IL-17, MIP-1α, PDGF-BB, VEGF, MCP-1, G-CSF, IFN-γ, eotaxin, and TNF-α) are significantly upregulated in the CSF of severe SMA1 patients compared to milder SMA2 and SMA3 patients, who display a range of values comparable to those found in the CSF of pediatric healthy subjects in the present work and in a previous study^28^. Among the cytokines upregulated in the CSF of SMA1 patients, we also found IL-4 that mainly acts as an anti-inflammatory secreted molecule^29^. IL-4 is a Th2-dependent cytokine that regulates both immune and neuronal functions^30^, and its selective increase in the CSF of naive SMA1 patients may reflect an attempt to counteract inflammation by activated Th2 cells.

The immune-modulatory effects of Nusinersen therapy in the CNS of SMA patients are unknown. Our longitudinal analysis shows that Nusinersen significantly reduces the CSF levels of several pro-inflammatory molecules (IL-2, IL-7, IL-9, IL-12, IL-17, VEGF, eotaxin and TNF-α) in SMA1 patients. These findings indicate that Nusinersen may counteract neuroinflammation in severe SMA infants. This possibility is also consistent with the observed normalization of IL-4 levels, which may reflect a generalized reduction in immune activation induced by Nusinersen in SMA1 patients. Moreover, since these effects are found after 302 but not 64 days of treatment, long-term Nusinersen therapy may be needed to effectively mitigate neuroinflammation in SMA. Importantly, however, Nusinersen does not attenuate the increased levels of other potent pro-inflammatory molecules (IL-6, IL-8, IFN-γ, MCP1, MIP-1α, and PDGF-BB), revealing that a subset of cytokines and neurotrophic factors is refractory to Nusinersen-dependent correction in SMA1 patients. This could originate from shortcomings of this CNS-restricted therapy in targeting specific cell types in the CNS or the lack of correction of peripheral immune system’s defects which have been documented in both SMA patients and mouse models^29,31^.

In this context, a recent study reported increased levels of various cytokines in the serum of adult and pediatric SMA patients and correction by Nusinersen after 180 days of treatment^29^, providing evidence for peripheral immune system dysfunction in the disease. However, analysis of the CSF of the same patients failed to identify clear signatures of neuroinflammation or modulation of central cytokine levels by Nusinersen^29^, which is seemingly inconsistent with our findings. This likely reflects differences in study design such as the shorter time of Nusinersen treatment (180 vs 302 days) and the use of a pooled cohort of SMA patients comprising different clinical types and the inclusion of only a handful of SMA1 patients which, in contrast to our stratified analysis by clinical type, may have hampered their identification of both baseline differences and Nusinersen-mediated changes in the CSF levels of inflammatory markers^29^. Nevertheless, their results also argue against blood brain barrier alterations and leakage of serum components to the CSF as key factors influencing the concentration of inflammatory markers in the CNS of SMA patients. Taken together, peripheral^29^ and central (this study) inflammation emerge as possibly distinct disease features of SMA patients, with neuroinflammation being specifically associated with severe SMA.

Our findings also highlight distinct effects of Nusinersen in the CSF of SMA2 and SMA3 patients, leading to the induction of the pro-inflammatory cytokines (IL-8, G-CSF, MCP-1, MIP-1α, and MIP-1β) or the decrease of a potent anti-inflammatory cytokine (IL-1ra), respectively. These neuroinflammatory outcomes may represent possible side-effects of Nusinersen in milder SMA patients. However, our results do not highlight a correlation between differences in the levels of these cytokines and specific changes in motor function of SMA2 and SMA3 patients assessed by the HFMSE. Considering also that significant adverse effects have yet to emerge following long-term treatment in milder SMA patients, the clinical relevance, if any, of the neuroinflammatory signatures elicited by Nusinersen in milder SMA patients remains to be established.

The cellular origin, underlying mechanisms, and clinical relevance of the neuroinflammatory alterations highlighted in our study require future investigation. However, the basal increase of pro-inflammatory cytokines such as IL-2, IL-12, IL-17, IFN-γ, and TNF-α in the CSF suggests activation of T-helper 1 (Th1)/Th17 cells in the CNS of SMA1 patients^27,32^. These immune cells are known to stimulate CNS-resident microglia and astrocytes that, in turn, can release potent pro-inflammatory molecules like TNF-α, IL-6 and MIP-1β under neurodegenerative conditions^33^. In support of a potential role for enhanced Th1/Th17 and microglia/astrocytes activation in promoting neuroinflammation in SMA1 patients, previous studies showed severe gliosis in spinal cord samples derived from patients^34–36^. Further supporting the existence of a link between neuroinflammation, gliosis and SMN protein deficiency, previous studies documented the activation of astrocytes and microglia as well as increased mRNA levels of pro-inflammatory cytokines (IL-1β, IL-6, and TNF-α) in the spinal cord and spleen of SMA mice^31^. Nevertheless, the origin of cytokine secretion remains to be established and its determination is beyond the scope of the present study.

Limitations of our study include the small sample size leading to a lack of gender- and age-matched controls for each clinical type of SMA patients as well as the absence of males in the cohort of SMA3 patients. Accordingly, only a limited number of CSF samples from pediatric non-neurological control subjects were available to us for this study. These were used as a reference for normal levels of cytokines concentrations but not included in the statistical analyses. As for the gender unbalance in the SMA3 cohort, we note that there is no established gender-specific difference in the clinical course of SMA. Moreover, the basal CSF levels of all inflammatory markers in samples from SMA3 patients do not differ from those measured in the samples from SMA2 patients, which comprised both males and females. Thus, whilst it is highly unlikely that the gender unbalance in the SMA3 group contributes to bias in the results, we cannot rule this out. Therefore, this result will need to be confirmed in a larger gender-matched study. It would also have been interesting to determine the state of peripheral inflammation in our cohort of SMA patients, but serum was not available for our retrospective analysis. Despite these limitations, we were able to identify significant increases in the CSF levels of many inflammatory cytokines in SMA1 patients relative to SMA2 and SMA3 patients through the application of stringent statistical analyses that accounted for the confounding factors between SMA groups. Furthermore, the conclusions from the longitudinal analysis of changes in cytokine levels induced by Nusinersen in the CSF of individual SMA patients are solid and independent from the availability of control groups. Nonetheless, future studies are warranted to confirm and expand our findings in larger cohorts of SMA patients and by using other analytical platforms.

In conclusion, our results reveal prominent signatures of neuroinflammation that are specifically associated with severe SMA and their partial suppression by Nusinersen therapy. They also suggest that pharmacological approaches addressing the outstanding signatures of neuroinflammation in Nusinersen-treated SMA patients may deserve consideration as candidate combination therapies. The development of targeted anti-inflammatory agents could help address limitations and further improve the clinical benefit of Nusinersen and possibly other SMN-enhancing treatments in SMA patients.

## Supplementary information

Description of Additional Supplementary Files

Supplementary Information

Supplementary Data 1

Reporting Summary

## Data Availability

The datasets generated during and/or analyzed during the current study are available from the corresponding author on reasonable request. Source data for the main figures in this manuscript are provided in excel format as Supplementary Data 1.

## References

[1] Lefebvre S (1995). Identification and characterization of a spinal muscular atrophy-determining gene. Cell.

[2] Tisdale S, Pellizzoni L (2015). Disease mechanisms and therapeutic approaches in spinal muscular atrophy. J. Neurosci..

[3] Wirth B, Karakaya M, Kye MJ, Mendoza-Ferreira N (2020). Twenty-five years of spinal muscular atrophy research: from phenotype to genotype to therapy, and what comes next. Annu. Rev. Genomics Hum. Genet..

[4] D’Amico A, Mercuri E, Tiziano FD, Bertini E (2011). Spinal muscular atrophy. Orphanet J. Rare Dis..

[5] Wirth B (2021). Spinal muscular atrophy: in the challenge lies a solution. Trends Neurosci..

[6] Ravi B, Chan-Cortes MH, Sumner CJ (2021). Gene-targeting therapeutics for neurological disease: lessons learned from spinal muscular atrophy. Annu. Rev. Med..

[7] Mercuri E, Pera MC, Scoto M, Finkel R, Muntoni F (2020). Spinal muscular atrophy - insights and challenges in the treatment era. Nat. Rev. Neurol..

[8] Finkel RS (2017). Nusinersen versus sham control in infantile-onset spinal muscular atrophy. N. Engl. J. Med..

[9] Finkel RS (2016). Treatment of infantile-onset spinal muscular atrophy with nusinersen: a phase 2, open-label, dose-escalation study. Lancet.

[10] Mercuri E (2018). Nusinersen versus sham control in later-onset spinal muscular atrophy. N. Engl. J. Med..

[11] Chen, X., Hu, Y., Cao, Z., Liu, Q. & Cheng, Y. Cerebrospinal Fluid inflammatory cytokine aberrations in Alzheimer’s disease, parkinson’s disease and amyotrophic lateral sclerosis: a systematic review and meta-analysis. *Front. Immunol.* (2018). 10.3389/fimmu.2018.02122 PMC6156158 30283455

[12] Glass CK, Saijo K, Winner B, Marchetto MC, Gage FH (2010). Mechanisms underlying inflammation in neurodegeneration. Cell.

[13] Mitchell RM (2008). A CSF biomarker panel for identification of patients with amyotrophic lateral sclerosis. Neurology.

[14] Abati E, Citterio G, Bresolin N, Comi GP, Corti S (2020). Glial cells involvement in spinal muscular atrophy: could SMA be a neuroinflammatory disease. Neurobiol. Dis..

[15] Deguise MO, Kothary R (2017). New insights into SMA pathogenesis: immune dysfunction and neuroinflammation. Ann. Clin. Transl. Neurol..

[16] Papadimitriou D (2010). Inflammation in ALS and SMA: sorting out the good from the evil. Neurobiol. Dis..

[17] Pane M (2019). Nusinersen in type 1 spinal muscular atrophy: Twelve-month real-world data. Ann. Neurol..

[18] Pane M (2018). An observational study of functional abilities in infants, children, and adults with type 1 SMA. Neurology.

[19] Coratti G (2021). Motor function in type 2 and 3 SMA patients treated with Nusinersen: a critical review and meta-analysis. Orphanet J. Rare Dis..

[20] Glanzman AM (2018). Evaluator training and reliability for SMA global nusinersen trials1. J. Neuromuscul. Dis..

[21] Glanzman AM (2010). The Children’s Hospital of Philadelphia Infant Test of Neuromuscular Disorders (CHOP INTEND): test development and reliability. Neuromuscul. Disord..

[22] Glanzman AM (2011). Validation of the Children’s Hospital of Philadelphia Infant Test of Neuromuscular Disorders (CHOP INTEND). Pediatr. Phys. Ther.

[23] O’Hagen JM (2007). An expanded version of the Hammersmith Functional Motor Scale for SMA II and III patients. Neuromuscul. Disord..

[24] Pieragostino D (2018). Enhanced release of acid sphingomyelinase-enriched exosomes generates a lipidomics signature in CSF of Multiple Sclerosis patients. Sci. Rep..

[25] Miceli M (2016). Secretome profiling of cytokines and growth factors reveals that neuro-glial differentiation is associated with the down-regulation of Chemokine Ligand 2 (MCP-1/CCL2) in amniotic fluid derived-mesenchymal progenitor cells. Proteomics.

[26] Kempuraj, D. et al. Neuroinflammation induces neurodegeneration. *J. Neurol. Neurosurg. Spine* 1, 1003 (2016). PMC5260818 28127589

[27] Becher B, Spath S, Goverman J (2017). Cytokine networks in neuroinflammation. Nat. Rev. Immunol..

[28] Pranzatelli MR, Tate ED, McGee NR, Colliver JA (2013). Pediatric reference ranges for proinflammatory and anti-inflammatory cytokines in cerebrospinal fluid and serum by multiplexed immunoassay. J. Interferon Cytokine Res..

[29] Bonanno S (2022). Identification of a cytokine profile in serum and cerebrospinal fluid of pediatric and adult spinal muscular atrophy patients and its modulation upon nusinersen treatment. Front. Cell Neurosci..

[30] Oetjen LK, Kim BS (2018). Interactions of the immune and sensory nervous systems in atopy. FEBS J..

[31] Deguise MO (2017). Immune dysregulation may contribute to disease pathogenesis in spinal muscular atrophy mice. Hum. Mol. Genet..

[32] Damsker JM, Hansen AM, Caspi RR (2010). Th1 and Th17 cells: adversaries and collaborators. Ann. N. Y. Acad. Sci..

[33] Smith JA, Das A, Ray SK, Banik NL (2012). Role of pro-inflammatory cytokines released from microglia in neurodegenerative diseases. Brain Res. Bull..

[34] Rindt H (2015). Astrocytes influence the severity of spinal muscular atrophy. Hum. Mol. Genet..

[35] Kuru, S. et al. An autopsy case of spinal muscular atrophy type III Kugelberg-Welander disease. *Neuropathology* **29**, 63–67 (2009). 10.1111/j.1440-1789.2008.00910.x 18410269

[36] Brock TO, McIlwain DL (1984). Astrocytic proteins in the dorsal and ventral roots in amyotrophic lateral sclerosis and Werdnig-Hoffmann disease. J. Neuropathol. Exp. Neurol..

