# Supplementary material for: Nusinersen mitigates neuroinflammation in severe spinal muscular atrophy patients
Source: Commun Med (Lond). 2023 Feb 15;3:28. doi: 10.1038/s43856-023-00256-2 (PMC9932014; doi:10.1038/s43856-023-00256-2)
Supplement: Supplementary file 2 — Supplementary Information [file 43856_2023_256_MOESM2_ESM.pdf]

Supplementary information for

Nusinersen mitigates neuroinflammation in severe Spinal Muscular Atrophy patients

Tommaso Nuzzo[1,2,*], Rosita Russo[1,*], Francesco Errico[2,3,*], Adele D'Amico[4,*], Awet G. Tewelde[1], Mariangela Valletta[1], Amber Hassan[2], Michele Tosi[4], Chiara Panicucci[5], Claudio Bruno[5,6], Enrico Bertini[4], Angela Chambery[1], Livio Pellizzoni[7,8,9], Alessandro Usiello[1,2,@]

[1]Department of Environmental, Biological and Pharmaceutical Science and Technologies, Università degli Studi della Campania "Luigi Vanvitelli", Caserta, Italy.
[2]Laboratory of Translational Neuroscience, Ceinge Biotecnologie Avanzate, Naples, Italy.
[3]Department of Agricultural Sciences, University of Naples "Federico II", Portici, Italy.
[4]Unit of Neuromuscular and Neurodegenerative Disorders, Dept. Neurosciences, Bambino Gesu' Children's Hospital IRCCS, Roma, Italy.
[5]Center of Translational and Experimental Myology, IRCCS Istituto Giannina Gaslini, Genova, Italy.
[6]Department of Neuroscience, Rehabilitation, Ophthalmology, Genetics, Maternal, and Child Health - DINOGMI, University of Genova
[7]Center for Motor Neuron Biology and Disease, Columbia University, New York, NY, USA.
[8]Department of Pathology and Cell Biology, Columbia University, New York, NY, USA.
[9]Department of Neurology, Columbia University, New York, NY, USA.

*These authors contributed equally.

[@]Corresponding Author:
Alessandro Usiello, Ph.D.: Department of Environmental, Biological and Pharmaceutical Sciences and Technologies, University of Campania "Luigi Vanvitelli", Via A. Vivaldi, 43, 81100 Caserta, Italy, and CEINGE Biotecnologie Avanzate, Naples, Italy; Phone: +39 0813737879, email: usiello@ceinge.unina.it; alessandro.usiello@unicampanina.it

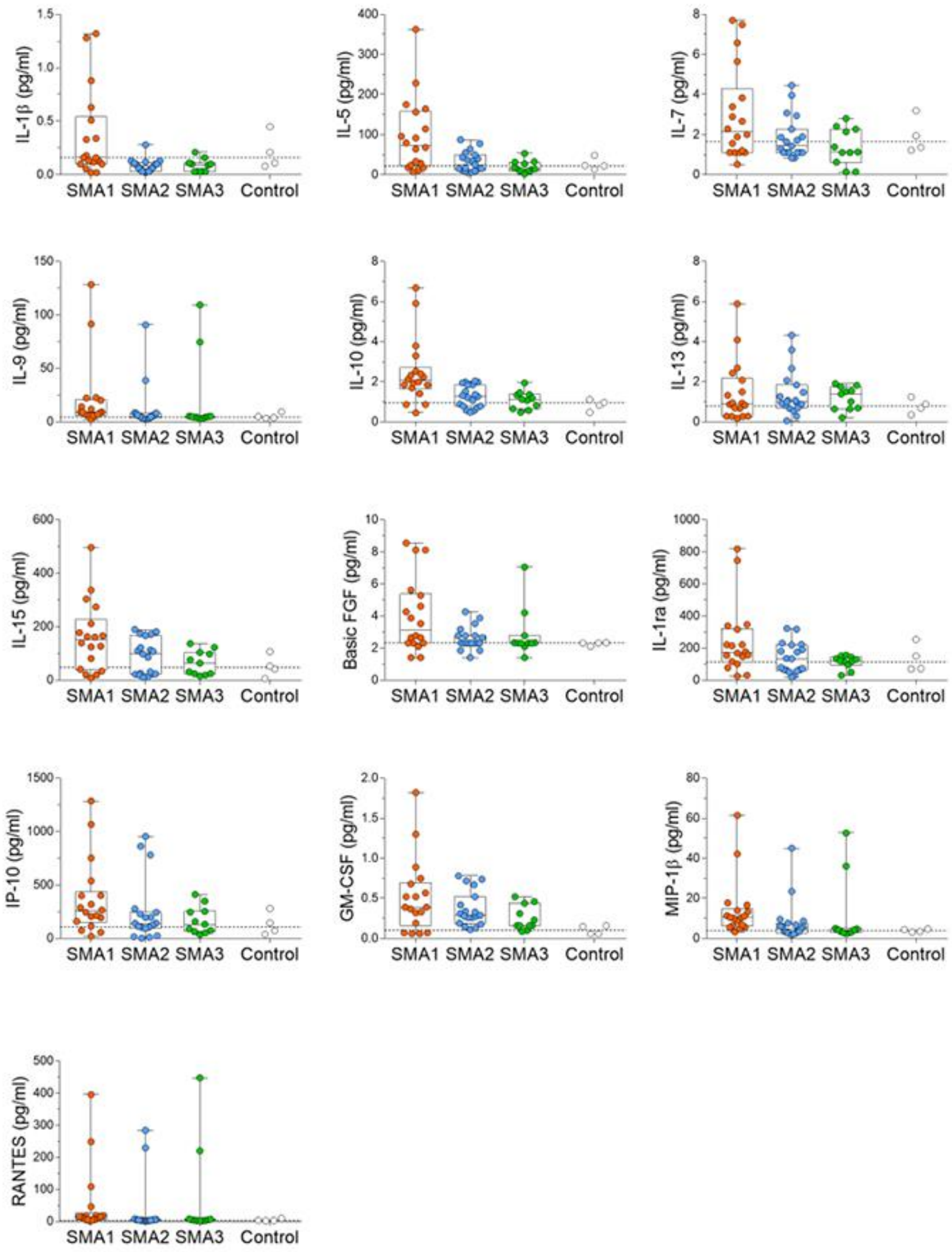

Supplementary Figure 1. *Levels of cytokines and neurotrophic factors that do not differ in the CSF of naïve SMA types 1, 2 and 3 patients.* CSF levels of interleukin (IL)-1β, IL-5, IL-7, IL-9, IL-10, IL-13, IL-15, basic fibroblast growth factor (FGF-basic), IL-1ra, IP-10, granulocyte macrophage colony stimulating factor (GM-CSF), macrophage inflammatory protein 1-beta (MIP-1β) and RANTES in untreated SMA1 (n=18), SMA2 (n=19) and SMA3 (n=11) patients. Controls (n=4) were used as a reference for physiological baseline levels. Data were analyzed by ANCOVA considering the effect of different factors (age, sex, BMI, *SMN2* copy number, gastrostomy, NIV, and tracheostomy). Data are shown as box and whisker plots representing median with interquartile range (IQR). Dots represent individual patients' values. The dotted line represents the median of control samples.

SMA1

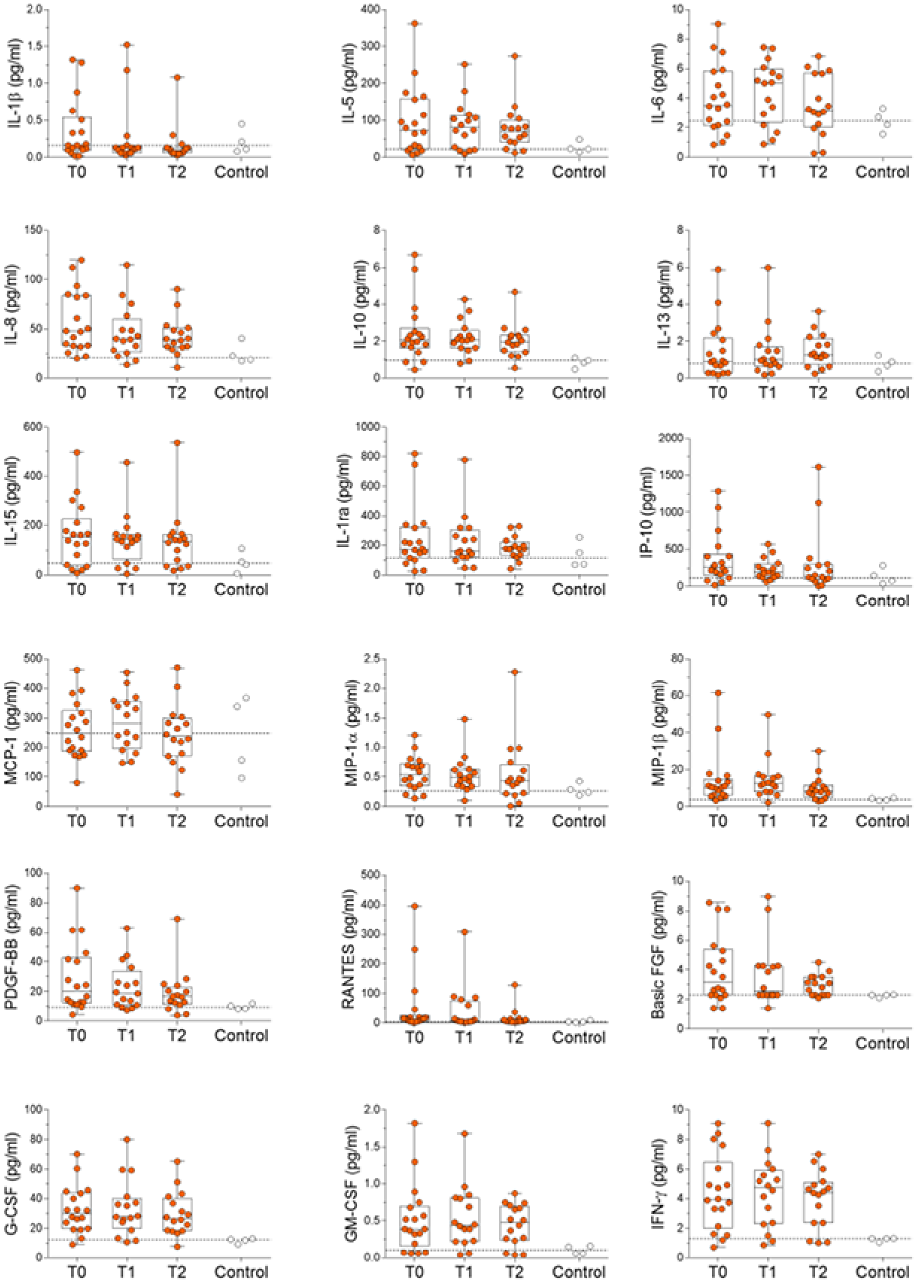

Supplementary Figure 2. *Levels of cytokines and neurotrophic factors that are unaffected by Nusinersen treatment in the CSF of SMA1 patients.* Box and whisker plots showing unaltered CSF levels of IL-1β, IL-5, IL-6, IL-8, IL-10, IL-13, IL-15, IL-1ra, IP-10, MCP-1, MIP-1α, MIP-1β, PDGF-BB, RANTES, FGF-basic, G-CSF, GM-CSF and IFN-γ in SMA1 patients prior to treatment (T0) and at the time of the fourth (T1) and sixth (T2) injection of Nusinersen. Controls (n=4) were used as a reference for physiological baseline levels of cytokines. Data are analyzed by Wilcoxon matched-pairs signed ranks test and shown as box and whisker plots representing median with interquartile range (IQR). Dots represent individual patients' values. The dotted line represents the median of control samples.

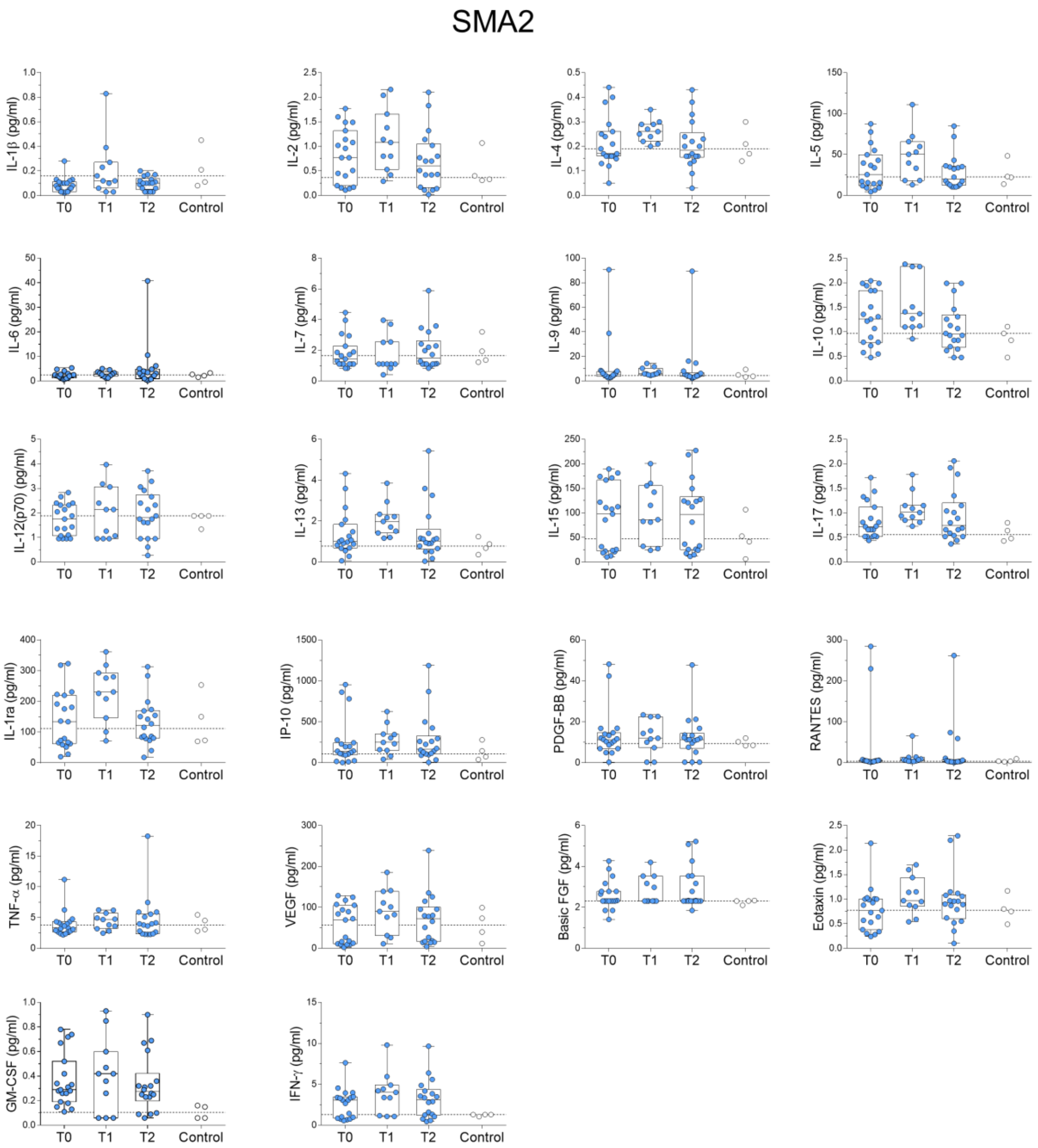

Supplementary Figure 3. *Levels of cytokines and neurotrophic factors that are unaltered by Nusinersen treatment in the CSF of SMA2 patients.* Unaltered CSF levels of interleukin IL-1β, IL-2, IL-4, IL-5, IL-6, IL-7, IL-9, IL-10, IL-12 (p70), IL-13, IL-15, IL-17A, IL-1ra, IP-10, PDGF-BB, RANTES, TNFα, VEGF, FGF-basic, Eotaxin, GM-CSF and IFN-γ cytokines in SMA2 patients prior to treatment (T0) and at T1 and T2 of Nusinersen treatment. Controls (n=4) were used as a reference for physiological baseline levels of cytokines. Data are analyzed by Wilcoxon matched-pairs signed ranks test and shown as box and whisker plots representing median with interquartile range (IQR). Dots represent individual patients' values. The dotted line represents the median of control samples.

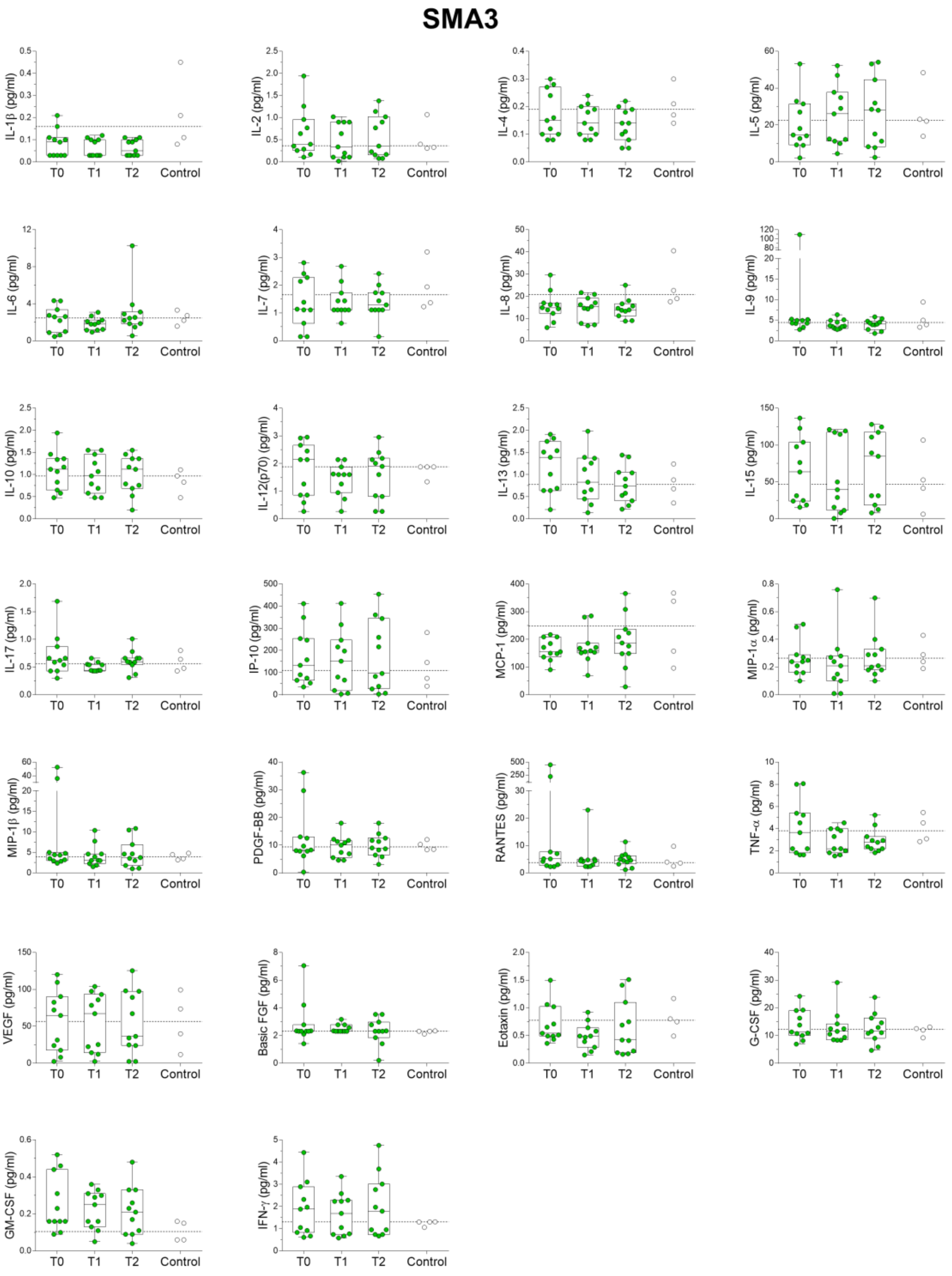

Supplementary Figure 4. *Levels of cytokines and neurotrophic factors that are unaltered by Nusinersen treatment in the CSF of SMA3 patients*. Box and whisker plots showing the CSF levels of IL-1β, IL-2, IL-4, IL-5, IL-6, IL-7, IL-8, IL-9, IL-10, IL-12 (p70), IL-13, IL-15, IL-17A, IP-10, MCP-1, MIP-1α, MIP-1β, PDGF-BB, RANTES, TNF-α, VEGF, FGF-basic, Eotaxin, G-CSF, GM-CSF, and IFN-γ in SMA3 patients prior to treatment (T0) and at the time of the fourth (T1) and sixth (T2) injection of Nusinersen. Controls (n=4) were used as a reference for physiological baseline levels of cytokines. Data are analyzed by Wilcoxon matched-pairs signed ranks test and shown as box and whisker plots representing median with interquartile range (IQR). Dots represent individual patients' values. The dotted line represents the median of control sample.

Supplementary Table 1. Clinical characteristics of SMA subjects enrolled in the study at fourth (T1) and sixth (T2) injection of Nusinersen.

SMA	Nusinersen	CHOP-INTEND		HFMSE		Gastrostomy		NIV		Tracheostomy		CSF total proteins (µg/µl)		CSF pH	
		n	*Median [Min;Max]*	*n*	*Median [Min;Max]*	*No*	*Yes*	*No*	*Yes*	*No*	*Yes*	*n*	*Median [Min;Max]*	*n*	*Median [Min;Max]*
1	T0	18	7.5[0;52]			4	14	12	6	10	8	18	0.18[0.077;0.981]	18	8.82[7.74;9.09]
	T1	14	9[0;64]			3	13	9	7	9	7	15	0.19[0.069;0.618]	15	8.82[8.44;9.34]
	T2	16	12[0;64]			3	13	9	7	9	7	16	0.23[0.124;0.541]	16	8.93[8.22;9.09]
2	T0			19	9[2;41]	19	0	12	7	19	0	19	0.18[0.121;0.497]	19	8.79[7.6;9.29]
	T1			7	7[2;23]	11	0	7	4	11	0	11	0.22[0.101;0.402]	11	8.98[7.61;9.27]
	T2			17	13[2;57]	18	0	12	6	18	0	16	0.22[0.084;0.825]	16	8.79[8.05;9.44]
3	T0			11	45[15;62]	11	0	10	1	11	0	11	0.18[0.044;0.65]	11	8.87[8.15;9.17]
	T1			9	39[17;62]	11	0	11	0	11	0	11	0.16[0.094;0.481]	11	8.82[8.52;9.17]
	T2			11	48[14;62]	11	0	10	1	11	0	11	0.14[0.045;0.481]	11	8.89[8.77;9.05]

Values are expressed as median (min max). For gastrostomy, tracheostomy and NIV, number of subjects (n) is indicated. Statistical analyses were performed by Mann-Whitney test. Abbreviations: CHOP-INTEND = Children's Hospital Of Philadelphia Infant Test Of Neuromuscular Disorders; HFMSE = Hammersmith Functional Motor Scale Expanded; NIV= Non-invasive ventilation; CSF = Cerebrospinal fluid.

Supplementary Table 2. Cerebrospinal fluid cytokines and neurotrophic factors levels (expressed as pg/ml) compared among the different naive SMA types.

	Control (n=4)	SMA1 (n=18)	SMA2 (n=19)	SMA3 (n=11)	ANCOVA		Bonferroni (*p* value)		
Analyte (pg/ml)	Median [Min;Max]	Median [Min;Max]	Median [Min;Max]	Median [Min;Max]	$F_{(2.44)}$	*p* value	SMA1 vs SMA2	SMA1 vs SMA3	SMA2 vs SMA3
Basic FGF	2.3[2.1;2.34]	3.15[1.41;8.55]	2.3[1.41;4.27]	2.3[1.41;7.05]	0.137	0.873	-	-	-
Eotaxin	0.77[0.49;1.17]	1.42[0.31;3.5]	0.77[0.24;2.14]	0.54[0.36;1.5]	9.764	**0.0004**	**0.040***	**0.005****	1.00
G-CSF	12.25[9.17;13.08]	29.79[9;70.33]	18.27[5.53;31.95]	11.28[6.93;24.18]	5.700	**0.007**	**0.0005****	**0.0002****	1.00
GM-CSF	0.1[0.05;0.16]	0.39[0.06;1.82]	0.29[0.11;0.78]	0.16[0.09;0.52]	3.124	0.056	-	-	-
IFN-γ	1.3[1.06;1.31]	3.93[0.71;9.09]	3.06[0.57;7.63]	1.88[0.61;4.43]	3.683	**0.035**	**0.032***	**0.007****	1.00
IL-10	0.9[0.48;1.11]	2.06[0.45;6.69]	1.26[0.48;2.04]	1.12[0.48;1.94]	2.786	0.075	-	-	-
IL-12(p70)	1.88[1.34;1.9]	3[0.86;11.2]	1.75[0.95;2.83]	2.14[0.27;2.95]	5.051	**0.012**	**0.003****	**0.011***	1.00
IL-13	0.78[0.35;1.23]	0.9[0.17;5.89]	1[0.06;4.32]	1.39[0.21;1.91]	0.548	0.583	-	-	-
IL-15	47.2[6.43;106.72]	150.11[8.16;496.74]	97.82[10.36;189.57]	63.5[15.83;136.62]	1.396	0.261	-	-	-
IL-17	0.64[0.43;1.98]	1.55[0.44;3.21]	0.72[0.44;1.72]	0.62[0.3;1.69]	6.809	**0.003**	**0.0007****	**0.0007****	1.00
IL-1β	0.15[0.08;0.45]	0.16[0.02;1.32]	0.08[0.02;0.28]	0.09[0.03;0.21]	0.723	0.492	-	-	-
IL-1ra	111.65[69.85;253.59]	173.13[25.52;818.72]	133.74[19.67;323.33]	120.33[30.69;156.32]	2.017	0.148	-	-	-
IL-2	0.36[0.31;1.07]	1.7[0.17;5.64]	0.77[0.11;1.77]	0.4[0.11;1.94]	3.320	**0.048**	**0.001****	**0.002****	1.00
IL-4	0.19[0.14;0.3]	0.25[0.03;0.73]	0.17[0.05;0.44]	0.15[0.08;0.3]	4.047	**0.026**	0.086	**0.029***	1.00
IL-5	22.64[13.94;48.38]	73.96[7.04;362.81]	25.57[4.53;87.56]	14.62[2.22;53.14]	2.395	0.106	-	-	-
IL-6	2.46[1.57;3.3]	3.51[0.85;9.04]	2.18[0.63;5.37]	2.58[0.45;4.33]	3.388	**0.045**	**0.019***	0.050	1.00
IL-7	1.65[1.23;3.2]	2.15[0.52;7.71]	1.44[0.84;4.46]	1.14[0.15;2.81]	1.146	0.329	-	-	-
IL-8	20.82[17.61;40.51]	47.67[20.24;120.04]	20.56[8.92;43.6]	14.91[5.95;29.62]	4.336	**0.021**	**<0.0001*****	**<0.0001*****	0.93
IL-9	4.44[3.31;9.43]	9.09[2.23;128.48]	5.1[2.61;90.72]	4.95[2.74;109.39]	1.075	0.352	-	-	-
IP-10	110.3[38.84;281.16]	255.71[20.47;1287.46]	141.45[3.47;955.22]	132.92[35.8;411.42]	6.986	**0.003**	0.586	0.236	1.00
MCP-1	248.02[96.51;367.49]	247.6[80.78;463.24]	168.61[95.19;477.39]	155.63[90.35;217.4]	4.673	**0.016**	0.104	**0.013***	0.79
MIP-1α	0.26[0.18;0.43]	0.53[0.14;1.21]	0.25[0.06;0.48]	0.24[0.1;0.51]	8.328	**0.001**	**0.0002****	**0.001****	1.00
MIP-1β	3.98[3.18;4.81]	10.44[3.32;61.43]	4.97[1.85;45.1]	4.34[2.35;52.63]	1.977	0.154	-	-	-
PDGF-BB	10.28[8.37;35.79]	20.14[4.28;89.97]	11.31[0.3;48.21]	8.29[0.3;36.29]	5.025	**0.012**	**0.027***	**0.044***	1.00
RANTES	3.77[2.49;9.75]	14.31[1.26;395.48]	4.77[1.83;284.35]	5.12[2.27;447.93]	0.724	0.492	-	-	-
TNF-α	3.8[2.83;5.45]	6.05[2.56;18]	3.37[2.19;11.2]	3.64[1.63;8.06]	4.396	**0.019**	**0.012***	**0.047***	1.00
VEGF	56.73[11.85;99.29]	124.11[2.37;370.44]	69.28[2.37;128.92]	64.74[2.37;120]	4.638	**0.016**	**0.003****	**0.011***	1.00

Values are expressed as median (min; max). Statistical analyses were performed by ANCOVA considering the effect of age, sex, BMI, SMN2 copy, gastrostomy, NIV/tracheostomy, followed by Bonferroni multiple comparisons when required. Significant *p* values are shown in bold (* $p<0.05$, ** $p<0.01$, *** $p<0.0001$).

Supplementary Table 3. Comparisons of cytokines and neurotrophic factors levels between subjects with or without gastrostomy, Non-invasive ventilation (NIV) or tracheostomy within each SMA type and treatment group. Uncorrected *p* values obtained by Mann-Whitney test are reported. All *p* values are not significant after correction with Benjamini-Hochberg multiple comparisons.

Analyte	SMA1									SMA2		
	T0	T1	T2	T0	T1	T2	T0	T1	T2	T0	T1	T2
	Gastrostomy			NIV			Tracheostomy			NIV		
	Yes vs No (p value)	*Yes vs No (p value)*	*Yes vs No (p value)*	*Yes vs No (p value)*	*Yes vs No (p value)*	*Yes vs No (p value)*	*Yes vs No (p value)*	*Yes vs No (p value)*	*Yes vs No (p value)*	*Yes vs No (p value)*	*Yes vs No (p value)*	*Yes vs No (p value)*
Basic FGF	0.873	0.944	0.583	0.511	0.406	0.215	0.448	0.956	0.388	0.515	0.918	0.845
Eotaxin	0.167	0.346	0.500	0.512	0.185	0.039	0.594	0.203	0.071	0.139	0.186	0.160
G-CSF	0.167	0.840	0.253	0.349	0.223	0.315	0.790	0.491	0.427	0.118	0.257	0.512
GM-CSF	0.264	0.501	0.946	1.000	0.397	0.634	0.350	0.491	0.791	0.767	0.704	0.851
IFN-γ	0.137	0.946	0.252	0.075	0.491	0.112	0.657	0.874	0.289	0.422	0.776	0.512
IL-10	0.832	0.545	0.736	0.606	0.427	0.596	0.756	0.185	0.289	0.472	0.184	0.091
IL-12(p70)	0.832	0.382	0.458	0.925	0.030	0.203	0.929	0.039	0.671	0.766	0.251	0.707
IL-13	0.288	0.637	0.946	0.425	0.266	0.711	0.894	0.314	0.491	0.472	0.571	0.888
IL-15	1.000	0.946	0.201	0.779	0.711	0.958	0.790	0.315	0.634	0.554	0.850	0.223
IL-17	0.222	0.382	0.543	0.174	0.289	0.457	0.790	0.340	0.671	0.832	0.344	0.399
IL-1β	0.831	0.172	0.945	0.963	0.485	0.554	0.894	0.237	0.872	0.831	0.296	0.538
IL-1ra	0.595	0.893	0.252	0.673	0.832	0.791	0.965	0.791	0.711	0.237	0.345	0.399
IL-2	0.750	0.736	0.736	0.574	0.958	0.671	0.424	0.711	0.791	0.310	0.185	0.281
IL-4	0.288	0.686	0.788	0.606	0.185	0.458	0.689	0.340	0.958	0.445	0.636	0.962
IL-5	0.671	0.840	0.840	0.925	0.958	0.315	0.657	0.427	0.223	0.352	0.185	0.134
IL-6	0.915	0.459	0.201	1.000	0.874	0.791	0.929	0.874	0.634	0.447	0.705	0.851
IL-7	0.240	0.946	0.456	0.962	0.138	0.165	0.304	0.525	0.262	0.932	0.552	0.344
IL-8	1.000	0.638	0.459	0.075	0.125	0.560	0.091	0.223	0.560	0.237	0.705	0.303
IL-9	0.243	0.638	0.946	0.111	0.186	0.491	0.594	0.368	0.958	0.220	0.131	0.039
IP-10	0.203	0.019	0.737	0.223	0.186	0.427	0.929	0.958	0.711	0.035	0.059	0.134
MCP-1	0.034	0.382	0.201	0.111	0.368	0.081	0.790	0.874	0.634	0.128	0.345	0.640
MIP-1α	0.111	0.122	0.106	0.512	0.090	0.916	0.477	0.289	0.634	0.108	0.131	0.399
MIP-1β	0.034	0.545	0.158	0.031	0.560	0.958	0.790	0.791	0.634	0.272	0.186	0.092
PDGF-BB	0.339	0.459	0.638	0.223	0.050	0.958	0.722	0.064	0.874	0.447	0.023	0.082
RANTES	0.034	0.382	0.638	0.160	0.223	0.368	0.657	0.266	0.634	0.057	0.008	0.039
TNF-α	0.202	0.313	0.840	0.325	0.101	0.153	0.894	0.101	0.368	0.446	0.186	0.453
VEGF	0.457	0.382	0.637	0.349	0.427	0.916	0.790	0.315	0.672	0.353	0.089	0.061

Supplementary Table 4. Correlation analysis of CSF cytokines and neurotrophic factors concentrations with age, BMI or CHOP-INTEND of SMA1 patients at first (T0), fourth (T1, loading) and sixth (T2, maintenance) injection of Nusinersen.

SMA1																											
Analyte	T0									T1									T2								
	Age			BMI			CHOP			Age			BMI			CHOP			Age			BMI			CHOP		
	r	*p value*	*n*	*r*	*p value*	*n*	*r*	*p value*	*n*	*r*	*p value*	*n*	*r*	*p value*	*n*	*r*	*p value*	*n*	*r*	*p value*	*n*	*r*	*p value*	*n*	*r*	*p value*	*n*
Basic FGF	-0.029	0.909	18	0.147	0.560	18	-0.136	0.591	18	-0.431	0.095	16	-0.070	0.837	11	0.202	0.489	14	-0.430	0.096	16	-0.286	0.321	14	0.255	0.340	16
Eotaxin	-0.156	0.537	18	0.309	0.213	18	0.164	0.516	18	-0.054	0.841	16	0.445	0.170	11	-0.180	0.538	14	-0.383	0.143	16	-0.165	0.572	14	0.389	0.137	16
G-CSF	-0.139	0.581	18	0.269	0.280	18	0.392	0.108	18	-0.035	0.897	16	0.027	0.937	11	0.007	0.982	14	-0.376	0.151	16	0.108	0.714	14	0.343	0.193	16
GM-CSF	0.192	0.444	18	0.042	0.867	18	0.214	0.395	18	0.094	0.729	16	0.300	0.370	11	0.042	0.887	14	0.106	0.696	16	0.055	0.852	14	-0.125	0.646	16
IFN-γ	-0.036	0.887	18	0.448	0.062	18	0.239	0.340	18	0.268	0.316	16	0.282	0.401	11	-0.380	0.181	14	-0.107	0.692	16	0.123	0.675	14	0.161	0.550	16
IL-10	0.137	0.587	18	-0.265	0.287	18	0.160	0.526	18	0.132	0.625	16	-0.055	0.873	11	0.241	0.407	14	0.219	0.415	16	-0.033	0.911	14	-0.173	0.521	16
IL-12(p70)	0.119	0.639	18	-0.071	0.779	18	0.121	0.631	18	-0.068	0.803	16	0.255	0.450	11	0.113	0.702	14	0.177	0.512	16	0.183	0.531	14	-0.229	0.394	16
IL-13	0.059	0.816	18	0.098	0.698	18	0.321	0.194	18	0.490	0.054	16	0.232	0.492	11	-0.170	0.561	14	0.168	0.535	16	-0.385	0.175	14	-0.044	0.871	16
IL-15	0.139	0.581	18	-0.024	0.926	18	0.085	0.737	18	0.318	0.231	16	-0.200	0.555	11	-0.031	0.916	14	0.409	0.116	16	-0.288	0.318	14	-0.138	0.609	16
IL-17	-0.007	0.977	18	0.347	0.158	18	0.179	0.477	18	0.041	0.880	16	0.465	0.150	11	-0.027	0.928	14	-0.306	0.250	16	0.152	0.603	14	0.208	0.440	16
IL-1β	0.042	0.867	18	0.017	0.948	18	-0.092	0.717	18	-0.223	0.407	16	-0.093	0.785	11	-0.059	0.842	14	-0.064	0.813	16	0.339	0.236	14	-0.059	0.828	16
IL-1ra	0.110	0.663	18	0.023	0.929	18	0.220	0.381	18	0.247	0.356	16	-0.050	0.884	11	-0.048	0.872	14	0.041	0.880	16	-0.240	0.409	14	-0.019	0.944	16
IL-2	-0.002	0.994	18	-0.160	0.526	18	0.288	0.246	18	0.141	0.602	16	0.050	0.884	11	-0.008	0.979	14	0.069	0.799	16	-0.051	0.863	14	-0.018	0.946	16
IL-4	-0.107	0.671	18	0.118	0.642	18	0.273	0.273	18	-0.119	0.660	16	-0.041	0.905	11	-0.033	0.910	14	-0.049	0.858	16	0.136	0.642	14	-0.119	0.661	16
IL-5	0.228	0.363	18	-0.143	0.570	18	0.057	0.822	18	0.218	0.418	16	-0.118	0.729	11	0.002	0.994	14	0.294	0.269	16	-0.015	0.958	14	-0.191	0.478	16
IL-6	0.214	0.395	18	-0.090	0.723	18	0.161	0.524	18	0.562	0.024	16	0.018	0.958	11	-0.064	0.828	14	0.241	0.368	16	-0.257	0.375	14	-0.010	0.970	16
IL-7	-0.330	0.181	18	0.194	0.441	18	0.158	0.530	18	-0.091	0.737	16	-0.018	0.958	11	-0.130	0.657	14	-0.193	0.474	16	-0.117	0.690	14	0.127	0.639	16
IL-8	-0.294	0.236	18	0.352	0.152	18	-0.179	0.476	18	0.041	0.880	16	-0.455	0.160	11	-0.325	0.258	14	0.094	0.729	16	0.011	0.970	14	0.003	0.991	16
IL-9	0.121	0.633	18	0.501	0.034	18	0.147	0.560	18	-0.203	0.451	16	0.445	0.170	11	0.121	0.679	14	0.082	0.762	16	-0.305	0.288	14	-0.090	0.741	16
IP-10	-0.251	0.316	18	0.385	0.115	18	0.251	0.315	18	-0.271	0.311	16	0.345	0.298	11	0.174	0.551	14	-0.274	0.305	16	-0.345	0.227	14	0.296	0.266	16
MCP-1	-0.251	0.316	18	0.674	**0.002**	18	0.272	0.275	18	0.144	0.594	16	0.518	0.102	11	-0.475	0.086	14	-0.126	0.641	16	0.130	0.659	14	0.166	0.538	16
MIP-1α	-0.337	0.171	18	0.311	0.210	18	0.349	0.156	18	-0.237	0.377	16	0.227	0.502	11	0.092	0.755	14	-0.673	0.004	16	0.000	1.000	14	0.575	0.020	16
MIP-1β	-0.127	0.616	18	0.717	**0.001**	18	0.251	0.315	18	-0.338	0.200	16	0.418	0.201	11	0.035	0.905	14	-0.553	0.026	16	-0.042	0.887	14	0.542	0.030	16
PDGF-BB	-0.194	0.440	18	0.264	0.289	18	0.023	0.928	18	-0.276	0.300	16	0.145	0.670	11	0.053	0.857	14	-0.206	0.444	16	0.266	0.358	14	0.034	0.901	16
RANTES	-0.186	0.460	18	0.680	**0.002**	18	0.266	0.286	18	-0.191	0.478	16	0.464	0.151	11	0.135	0.646	14	0.153	0.572	16	-0.332	0.246	14	0.032	0.905	16
TNF-α	-0.128	0.613	18	0.364	0.137	18	0.088	0.729	18	-0.315	0.235	16	0.236	0.484	11	-0.035	0.905	14	-0.305	0.251	16	0.240	0.409	14	0.185	0.493	16
VEGF	0.221	0.378	18	0.309	0.213	18	0.111	0.662	18	0.179	0.506	16	0.382	0.247	11	0.152	0.603	14	0.193	0.474	16	-0.002	0.994	14	0.026	0.924	16

Statistical analyses were performed by Spearman's correlations. *p* values that remain significant after correction with Benjamini-Hochberg multiple comparisons are shown in bold (see main text for *p* values after Benjamini-Hochberg correction).

Supplementary Table 5. Correlation analysis between CSF cytokines and neurotrophic factors concentrations and age, BMI or HFMSE of SMA2 patients at first (T0), fourth (T1, loading) and sixth (T2, maintenance) injection of Nusinersen.

	SMA2																										
Analyte	T0									T1									T2								
	Age			BMI			HFMSE			Age			BMI			HFMSE			Age			BMI			HFMSE		
	r	*p value*	*n*	*r*	*p value*	*n*	*r*	*p value*	*n*	*r*	*p value*	*n*	*r*	*p value*	*n*	*r*	*p value*	*n*	*r*	*p value*	*n*	*r*	*p value*	*n*	*r*	*p value*	*n*
Basic FGF	-0.099	0.686	19	-0.432	0.083	17	-0.190	0.437	19	0.702	0.016	11	0.200	0.747	5	-0.624	0.134	7	0.152	0.546	18	-0.089	0.762	14	-0.196	0.451	17
Eotaxin	0.338	0.157	19	-0.200	0.442	17	-0.271	0.262	19	0.323	0.332	11	-0.100	0.873	5	-0.595	0.159	7	0.171	0.496	18	0.092	0.753	14	-0.266	0.302	17
G-CSF	0.076	0.756	19	-0.334	0.191	17	-0.224	0.357	19	-0.173	0.611	11	-0.700	0.188	5	-0.180	0.699	7	-0.190	0.450	18	0.196	0.503	14	0.016	0.952	17
GM-CSF	0.002	0.994	19	-0.434	0.082	17	-0.094	0.701	19	0.128	0.707	11	-0.359	0.553	5	-0.573	0.179	7	0.052	0.838	18	0.135	0.647	14	-0.194	0.455	17
IFN-γ	-0.125	0.611	19	-0.224	0.387	17	-0.167	0.493	19	-0.064	0.852	11	-0.564	0.322	5	-0.200	0.667	7	-0.214	0.394	18	0.187	0.523	14	0.020	0.940	17
IL-10	-0.082	0.738	19	-0.274	0.288	17	-0.398	0.092	19	0.041	0.904	11	-0.462	0.434	5	-0.382	0.398	7	0.155	0.539	18	0.258	0.373	14	-0.276	0.283	17
IL-12(p70)	0.049	0.844	19	0.101	0.699	17	-0.188	0.440	19	0.567	0.069	11	0.200	0.747	5	-0.432	0.333	7	0.215	0.391	18	0.024	0.935	14	-0.325	0.203	17
IL-13	0.063	0.798	19	-0.514	0.035	17	-0.146	0.550	19	0.091	0.790	11	-0.500	0.391	5	-0.739	0.058	7	0.073	0.772	18	0.433	0.122	14	-0.054	0.837	17
IL-15	-0.130	0.596	19	-0.373	0.141	17	-0.270	0.263	19	-0.337	0.311	11	-0.600	0.285	5	-0.090	0.848	7	0.098	0.699	18	0.363	0.203	14	-0.317	0.215	17
IL-17	0.103	0.674	19	-0.403	0.109	17	-0.284	0.239	19	0.142	0.678	11	-0.359	0.553	5	-0.355	0.435	7	0.073	0.772	18	0.029	0.923	14	-0.220	0.396	17
IL-1β	0.191	0.433	19	-0.026	0.921	17	-0.146	0.550	19	0.087	0.799	11	-0.103	0.870	5	0.436	0.328	7	0.147	0.560	18	0.172	0.556	14	-0.381	0.131	17
IL-1ra	0.090	0.713	19	-0.343	0.178	17	-0.224	0.357	19	-0.328	0.325	11	-0.700	0.188	5	-0.018	0.969	7	-0.060	0.813	18	0.341	0.233	14	-0.108	0.680	17
IL-2	-0.095	0.698	19	-0.237	0.360	17	-0.204	0.403	19	0.082	0.810	11	-0.300	0.624	5	-0.342	0.452	7	0.029	0.908	18	0.077	0.794	14	-0.175	0.501	17
IL-4	0.234	0.336	19	-0.331	0.195	17	-0.241	0.321	19	0.158	0.644	11	-0.900	0.037	5	-0.685	0.090	7	0.133	0.599	18	-0.204	0.483	14	-0.141	0.590	17
IL-5	0.018	0.943	19	-0.199	0.445	17	-0.213	0.382	19	-0.119	0.728	11	-0.462	0.434	5	-0.218	0.638	7	0.109	0.666	18	0.358	0.208	14	-0.250	0.332	17
IL-6	0.075	0.762	19	-0.306	0.232	17	-0.234	0.334	19	0.155	0.649	11	0.000	1.000	5	-0.126	0.788	7	0.159	0.529	18	-0.033	0.911	14	-0.098	0.708	17
IL-7	0.192	0.432	19	0.280	0.277	17	-0.034	0.889	19	0.143	0.674	11	-0.200	0.747	5	-0.036	0.938	7	-0.220	0.380	18	-0.576	0.031	14	0.159	0.543	17
IL-8	0.198	0.416	19	0.010	0.970	17	-0.122	0.617	19	-0.050	0.884	11	-0.400	0.505	5	-0.054	0.908	7	-0.118	0.642	18	0.204	0.483	14	-0.139	0.595	17
IL-9	0.296	0.219	19	-0.259	0.316	17	-0.401	0.089	19	0.050	0.884	11	-0.500	0.391	5	-0.414	0.355	7	0.176	0.486	18	0.260	0.370	14	-0.231	0.372	17
IP-10	0.526	0.021	19	-0.059	0.823	17	-0.285	0.236	19	0.383	0.245	11	0.100	0.873	5	-0.324	0.478	7	0.164	0.515	18	0.138	0.637	14	-0.238	0.357	17
MCP-1	0.225	0.355	19	0.027	0.918	17	-0.063	0.799	19	0.091	0.790	11	-0.100	0.873	5	-0.270	0.558	7	-0.093	0.714	18	0.042	0.887	14	-0.028	0.914	17
MIP-1α	0.272	0.259	19	-0.177	0.498	17	-0.245	0.313	19	0.187	0.582	11	-0.400	0.505	5	-0.324	0.478	7	-0.117	0.645	18	0.165	0.573	14	0.049	0.852	17
MIP-1β	0.218	0.371	19	-0.419	0.094	17	-0.322	0.179	19	-0.018	0.958	11	-0.200	0.747	5	-0.162	0.728	7	0.068	0.788	18	0.244	0.401	14	-0.253	0.327	17
PDGF-BB	-0.040	0.872	19	-0.184	0.480	17	-0.136	0.578	19	0.475	0.140	11	0.600	0.285	5	-0.577	0.175	7	0.662	0.003	18	0.388	0.171	14	-0.720	0.001	17
RANTES	0.440	0.059	19	-0.076	0.772	17	-0.466	0.044	19	0.364	0.270	11	0.700	0.188	5	-0.396	0.379	7	0.285	0.252	18	0.121	0.681	14	-0.250	0.332	17
TNF-α	0.326	0.173	19	-0.041	0.877	17	-0.363	0.127	19	0.269	0.424	11	-0.200	0.747	5	-0.270	0.558	7	0.248	0.320	18	-0.161	0.583	14	-0.255	0.324	17
VEGF	0.134	0.584	19	-0.426	0.088	17	-0.213	0.381	19	-0.032	0.926	11	0.200	0.747	5	-0.180	0.699	7	0.232	0.354	18	0.292	0.311	14	-0.431	0.084	17

Statistical analyses were performed by Spearman's correlations. All *p* values are not significant after correction with Benjamini-Hochberg multiple comparisons.

Supplementary Table 6. Correlation analysis between CSF cytokines and neurotrophic factors concentrations and age, BMI or HFMSE of SMA3 patients at first (T0), fourth (T1, loading) and sixth (T2, maintenance) injection of Nusinersen.

SMA3																											
Analyte	T0									T1									T2								
	Age			BMI			HFMSE			Age			BMI			HFMSE			Age			BMI			HFMSE		
	r	*p value*	*n*	*r*	*p value*	*n*	*r*	*p value*	*n*	*r*	*p value*	*n*	*r*	*p value*	*n*	*r*	*p value*	*n*	*r*	*p value*	*n*	*r*	*p value*	*n*	*r*	*p value*	*n*
Basic FGF	-0.065	0.849	11	-0.172	0.634	10	0.224	0.507	11	0.323	0.333	11	0.078	0.831	10	0.090	0.818	9	0.221	0.514	11	-0.005	0.989	11	0.234	0.488	11
Eotaxin	0.027	0.936	11	-0.012	0.973	10	0.272	0.419	11	0.538	0.088	11	-0.116	0.751	10	-0.227	0.557	9	0.027	0.937	11	-0.327	0.326	11	0.009	0.979	11
G-CSF	-0.191	0.574	11	0.042	0.907	10	0.141	0.679	11	0.087	0.800	11	-0.220	0.542	10	-0.148	0.705	9	-0.245	0.467	11	-0.009	0.979	11	-0.227	0.502	11
GM-CSF	-0.489	0.127	11	-0.006	0.987	10	0.145	0.672	11	-0.487	0.128	11	0.043	0.907	10	0.441	0.235	9	-0.690	0.019	11	-0.192	0.572	11	0.142	0.678	11
IFN-γ	-0.309	0.355	11	0.127	0.726	10	-0.005	0.989	11	-0.292	0.384	11	-0.249	0.487	10	-0.361	0.339	9	-0.484	0.131	11	-0.237	0.482	11	-0.237	0.482	11
IL-10	-0.482	0.133	11	0.139	0.701	10	0.214	0.527	11	-0.178	0.600	11	0.159	0.662	10	0.239	0.535	9	-0.137	0.689	11	-0.059	0.863	11	0.255	0.449	11
IL-12(p70)	-0.333	0.316	11	-0.372	0.290	10	0.735	0.010	11	0.166	0.627	11	0.609	0.061	10	0.291	0.448	9	0.105	0.759	11	-0.100	0.769	11	0.333	0.318	11
IL-13	-0.082	0.811	11	0.067	0.855	10	0.443	0.172	11	0.091	0.790	11	0.188	0.603	10	-0.042	0.915	9	-0.227	0.502	11	0.173	0.612	11	-0.345	0.298	11
IL-15	-0.473	0.142	11	-0.018	0.960	10	-0.118	0.729	11	-0.018	0.958	11	0.200	0.580	10	-0.008	0.983	9	-0.323	0.332	11	0.059	0.863	11	-0.014	0.968	11
IL-17	-0.178	0.600	11	0.470	0.171	10	-0.085	0.805	11	0.506	0.112	11	0.411	0.238	10	-0.056	0.887	9	-0.464	0.150	11	-0.414	0.206	11	0.101	0.767	11
IL-1β	0.191	0.573	11	-0.100	0.783	10	0.316	0.344	11	0.283	0.399	11	0.285	0.424	10	0.000	1.000	9	0.259	0.442	11	-0.057	0.867	11	-0.144	0.673	11
IL-1ra	-0.018	0.958	11	0.681	0.030	10	-0.187	0.581	11	-0.096	0.779	11	0.427	0.219	10	-0.325	0.394	9	-0.118	0.729	11	0.173	0.612	11	-0.018	0.958	11
IL-2	-0.455	0.160	11	0.018	0.960	10	-0.178	0.601	11	-0.111	0.745	11	0.106	0.772	10	0.217	0.576	9	-0.351	0.290	11	-0.164	0.630	11	0.164	0.630	11
IL-4	0.187	0.581	11	0.073	0.841	10	0.391	0.234	11	-0.151	0.658	11	0.073	0.841	10	-0.080	0.838	9	0.352	0.289	11	-0.009	0.979	11	0.041	0.905	11
IL-5	-0.509	0.110	11	-0.103	0.777	10	-0.118	0.729	11	-0.209	0.537	11	0.164	0.651	10	0.100	0.797	9	-0.127	0.709	11	0.127	0.709	11	0.027	0.937	11
IL-6	0.191	0.574	11	0.188	0.603	10	0.191	0.573	11	0.137	0.689	11	0.097	0.789	10	0.201	0.604	9	-0.064	0.853	11	0.009	0.979	11	-0.255	0.450	11
IL-7	0.110	0.748	11	0.036	0.920	10	0.236	0.485	11	0.282	0.401	11	0.138	0.704	10	0.041	0.916	9	0.200	0.555	11	-0.209	0.537	11	-0.135	0.692	11
IL-8	0.309	0.355	11	0.345	0.328	10	-0.369	0.264	11	0.082	0.811	11	-0.248	0.489	10	-0.460	0.213	9	0.027	0.937	11	-0.291	0.385	11	-0.418	0.201	11
IL-9	-0.073	0.831	11	0.576	0.082	10	-0.050	0.883	11	0.036	0.915	11	0.285	0.425	10	-0.025	0.949	9	-0.236	0.484	11	-0.091	0.790	11	-0.091	0.790	11
IP-10	0.209	0.537	11	-0.176	0.627	10	0.433	0.184	11	0.418	0.201	11	-0.224	0.533	10	-0.276	0.472	9	-0.145	0.670	11	-0.145	0.670	11	-0.400	0.223	11
MCP-1	-0.027	0.937	11	-0.261	0.467	10	0.442	0.174	11	-0.018	0.958	11	-0.261	0.467	10	-0.561	0.116	9	-0.318	0.340	11	-0.264	0.433	11	-0.473	0.142	11
MIP-1α	0.329	0.324	11	-0.116	0.750	10	0.547	0.082	11	0.524	0.098	11	-0.286	0.424	10	-0.248	0.520	9	0.137	0.688	11	-0.169	0.619	11	-0.183	0.591	11
MIP-1β	-0.009	0.979	11	0.309	0.385	10	-0.205	0.545	11	0.355	0.285	11	0.079	0.829	10	-0.594	0.092	9	0.018	0.958	11	0.100	0.770	11	-0.436	0.180	11
PDGF-BB	-0.401	0.222	11	-0.200	0.580	10	-0.142	0.678	11	0.210	0.536	11	0.225	0.532	10	0.033	0.932	9	0.227	0.502	11	0.182	0.593	11	0.336	0.312	11
RANTES	-0.027	0.937	11	0.358	0.310	10	-0.392	0.233	11	0.105	0.758	11	0.275	0.441	10	-0.038	0.923	9	-0.145	0.670	11	-0.018	0.958	11	-0.273	0.417	11
TNF-α	0.100	0.769	11	-0.012	0.973	10	0.419	0.200	11	0.530	0.094	11	0.231	0.521	10	0.371	0.325	9	0.064	0.852	11	-0.279	0.407	11	0.068	0.841	11
VEGF	-0.336	0.312	11	0.067	0.855	10	0.123	0.719	11	-0.018	0.958	11	0.139	0.701	10	-0.151	0.699	9	-0.096	0.780	11	0.059	0.863	11	0.178	0.601	11

Statistical analyses were performed by Spearman's correlations. All *p* values are not significant after correction with Benjamini-Hochberg multiple comparisons.

Supplementary Table 7. CSF cytokines and neurotrophic factors levels (expressed as pg/ml) at T0 compared with T1 or T2 within each SMA1, SMA2 or SMA3 groups.

Analyte	SMA1					SMA2					SMA3				
	T0 (n=18)	T1 (n=16)	T2 (n=16)	T0 vs T1	T0 vs T2	T0 (n=19)	T1 (n=11)	T2 (n=18)	T0 vs T1	T0 vs T2	T0 (n=11)	T1 (n=11)	T2 (n=11)	T0 vs T1	T0 vs T2
	Median [Min;Max] (pg/ml)			*p value*		*Median [Min;Max] (pg/ml)*			*p value*		*Median [Min;Max] (pg/ml)*			*p value*	
Basic FGF	3.15[1.41;8.55]	2.54[1.41;8.96]	2.97[2.1;4.51]	0.35	0.118	2.3[1.41;4.27]	2.3[2.3;4.2]	2.32[1.85;5.22]	0.892	0.374	2.3[1.41;7.05]	2.3[2.3;3.17]	2.3[0.21;3.53]	0.753	0.342
Eotaxin	1,42[0,31;3,5]	0.97[0.5;3.28]	0.93[0.33;1,7]	0,125	0.017*	0,77[0,24;2,14]	0.97[0.54;1.7]	0.93[0.1;2.29]	0.646	0.239	0,54[0,36;1,5]	0.49[0.15;0.92]	0.42[0.16;1.51]	0.213	0.624
G-CSF	29.79[9;70.33]	27.97[10.7;80.04]	26.61[7.62;65.31]	0.795	0.214	18.27[5.53;31.95]	29.65[10.02;47.98]	21.37[4.12;50.78]	0.026*	0.027*	11.28[6.93;24.18]	11.52[8.31;29.15]	11.52[4.65;23.82]	0.372	0.373
GM-CSF	0.39[0.06;1.82]	0.44[0.04;1.68]	0.47[0.04;0.87]	0.733	0.334	0.29[0.11;0.78]	0.42[0.06;0.93]	0.27[0.06;0.9]	0.929	0.849	0.16[0.09;0.52]	0.25[0.05;0.36]	0.21[0.04;0.48]	0.414	0.504
IFN-γ	3.93[0.71;9.09]	4.71[0.86;9.1]	4.43[1.02;7.01]	0.501	0.099	3.06[0.57;7.63]	4.02[1.08;9.81]	3.12[0.46;9.66]	0.075	0.222	1.88[0.61;4.43]	1.69[0.58;3.35]	1.79[0.67;4.75]	0.656	0.789
IL-10	2.06[0.45;6.69]	2.09[0.79;4.27]	1.99[0.55;4.66]	0.736	0.111	1.26[0.48;2.04]	1.37[0.86;2.38]	0.95[0.48;1.99]	0.688	0.055	1.12[0.48;1.94]	0.97[0.48;1.55]	1.12[0.2;1.55]	0.259	0.964
IL-12(p70)	3[0.86;11.2]	2.76[0.86;6.61]	2.69[0.27;4.3]	0.211	0.035	1.75[0.95;2.83]	2.14[0.95;3.97]	1.81[0.27;3.71]	0.905	0.571	2.14[0.27;2.95]	1.6[0.27;2.14]	1.9[0.27;2.95]	0.332	0.767
IL-13	0.9[0.17;5.89]	0.99[0.18;5.99]	1.28[0.24;3.62]	0.856	0.876	1[0.06;4.32]	1.98[1.17;3.85]	1.02[0.04;5.43]	0.328	0.743	1.39[0.21;1.91]	0.83[0.14;1.98]	0.74[0.22;1.44]	0.286	0.075
IL-15	150.11[8.16;496.74]	143.6[4.89;455.81]	135.31[17.81;535.61]	0.379	0.070	97.82[10.36;189.57]	86.45[24.14;200.22]	96.97[11.53;227.24]	0.328	0.156	63.5[15.83;136.62]	39.52[0.69;121]	84.76[8.16;128.09]	0.13	0.373
IL-17	1.55[0.44;3.21]	1.36[0.52;3.19]	0.96[0.52;2.07]	0.569	0.004**	0.72[0.44;1.72]	1.01[0.73;1.78]	0.74[0.37;2.06]	0.838	0.277	0.62[0.3;1.69]	0.44[0.43;0.66]	0.6[0.31;1.01]	0.068	0.646
IL-1β	0.16[0.02;1.32]	0.12[0.03;1.52]	0.09[0.03;1.08]	0.155	0.055	0.08[0.02;0.28]	0.12[0.03;0.83]	0.1[0.03;0.2]	0.168	0.254	0.09[0.03;0.21]	0.03[0.03;0.12]	0.05[0.03;0.11]	0.197	0.293
IL-1ra	173.13[25.52;818.72]	162.23[47.87;777.72]	177.02[41.91;328.29]	0.795	0.255	133.74[19.67;323.33]	230.85[71.65;361.65]	121.39[17.7;312.99]	0.059	0.711	120.33[30.69;156.32]	78.64[49.77;173.53]	76.17[8.06;113.27]	0.13	0.02*
IL-2	1.7[0.17;5.64]	1.49[0.11;4.64]	1.6[0.05;3.24]	0.277	0.03*	0.77[0.11;1.77]	1.08[0.29;2.16]	0.6[0.02;2.1]	0.929	0.172	0.4[0.11;1.94]	0.34[0.02;1.02]	0.36[0.08;1.38]	0.091	0.384
IL-4	0.25[0.03;0.73]	0.25[0.09;0.74]	0.21[0.12;0.41]	0.756	0.046*	0.17[0.05;0.44]	0.26[0.2;0.35]	0.18[0.03;0.43]	0.858	0.827	0.15[0.08;0.3]	0.14[0.08;0.24]	0.14[0.05;0.22]	0.442	0.182
IL-5	73.96[7.04;362.81]	80.76[9.77;251.47]	69.34[12.23;273.88]	0.277	0.147	25.57[4.53;87.56]	50.27[13.2;111.04]	20.1[10.51;84.76]	0.213	0.831	14.62[2.22;53.14]	26.06[4.53;52.18]	28.02[2.48;54.09]	0.247	0.213
IL-6	3.51[0.85;9.04]	5.04[0.89;7.44]	3.17[0.27;6.84]	0.756	0.234	2.18[0.63;5.37]	2.76[1.28;5.07]	2.73[0.27;40.78]	0.398	0.17	2.58[0.45;4.33]	1.79[0.93;3.07]	2.43[0.53;10.26]	0.373	0.964
IL-7	2.15[0.52;7.71]	2.67[0.41;9.25]	1.37[0.41;4.7]	0.82	0.046*	1.44[0.84;4.46]	1.1[0.41;3.96]	1.51[0.84;5.89]	0.623	0.833	1.14[0.15;2.81]	1.1[0.63;2.68]	1.29[0.15;2.42]	0.678	0.858
IL-8	47.67[20.24;120.04]	39.85[13.5;114.98]	39.32[10.75;90.14]	0.408	0.108	20.56[8.92;43.6]	28.14[13.79;89.48]	23.37[12.63;57.02]	0.012*	0.306	14.91[5.95;29.62]	15.39[6.79;21.7]	13.89[8.86;25]	0.789	1,000
IL-9	9.09[2.23;128.48]	10.27[3.25;92.17]	7.25[2.68;32.78]	1,000	0.04*	5.1[2.61;90.72]	5.93[4.57;14.38]	4.57[2.17;89.44]	0.929	0.913	4.95[2.74;109.39]	3.56[2.74;6.33]	4.06[1.78;5.83]	0.085	0.114
IP-10	255.71[20.47;1287.46]	185.81[62.33;569.24]	137.69[4.95;1614.77]	0.178	0.147	141.45[3.47;955.22]	249.22[42.84;625.33]	165.4[5.86;1187.67]	0.593	0.093	132.92[35.8;411.42]	151.96[2.99;412.55]	96.31[2.99;454.17]	0.858	0.858
MCP-1	247.6[80.78;463.24]	280.55[148.02;454.25]	237.36[41.43;470.74]	0.108	0.534	168.61[95.19;477.39]	233.08[178.64;614.36]	274.76[29.5;707.81]	0.109	0.003**	155.63[90.35;217.4]	158.76[70.17;284.95]	187.14[28.92;365.83]	0.593	0.476
MIP-1α	0.53[0.14;1.21]	0.49[0.1;1.48]	0.44[0.01;2.28]	0.187	0.224	0.25[0.06;0.48]	0.31[0.02;1.01]	0.4[0.02;0.84]	0.307	0.027*	0.24[0.1;0.51]	0.21[0.01;0.76]	0.21[0.1;0.7]	0.929	0.929
MIP-1β	10.44[3.32;61.43]	12.3[2.15;49.67]	8.22[2.91;30.16]	0.338	0.301	4.97[1.85;45.1]	7.96[3.43;18.24]	8.63[1.01;43.08]	0.533	0.024*	4.34[2.35;52.63]	3.08[1.51;10.37]	3.75[1.01;10.85]	0.182	0.328
PDGF-BB	20.14[4.28;89.97]	19.16[7.38;62.77]	16.65[3.9;69.23]	0.393	0.07	11.31[0.3;48.21]	12.01[0.3;23.45]	11.32[0.3;47.82]	0.332	1,000	8.29[0.3;36.29]	10.01[4.59;17.98]	9.02[3.09;17.98]	0.373	0.789
RANTES	14.31[1.26;395.48]	7.63[2.22;307.87]	6.46[3.65;129.9]	0.437	0.087	4.77[1.83;284.35]	7.39[3.34;65.51]	3.37[0.74;262.16]	0.722	0.868	5.12[2.27;447.93]	4.39[2.27;23.03]	4.57[1.15;11.36]	0.213	0.286
TNF-α	6,05[2,56;18]	5.1[2.74;25.23]	3.63[2.19;8.34]	0,796	0,010*	3,37[2,19;11,2]	4.69[2.47;6.26]	3.9[2.28;18.28]	0.374	0.379	3,64[1,63;8,06]	2.19[1.53;4.52]	2.76[1.82;5.22]	0.091	0.384
VEGF	124.11[2.37;370.44]	105.66[2.25;299.99]	99.38[8.52;347.51]	0.795	0.026*	69.28[2.37;128.92]	90.03[11.13;185.62]	71.88[4.29;239.05]	0.476	0.239	64.74[2.37;120]	67.23[2.37;103.93]	36.45[2.37;125.36]	0.247	0.959

Values are expressed as median (min; max). Statistical analyses were performed by non-parametric Wilcoxon matched-pairs signed ranks. Significant *p* values are also marked with asterisks (* *p*<0.05, ***p*<0.01).

Supplementary Table 8. Comparisons of cytokines and neurotrophic factors levels between subjects that are responsive and not responsive to Nusinersen within each SMA type and treatment group. Uncorrected *p* values obtained by Mann-Whitney test are reported. All *p* values are not significant after correction with Benjamini-Hochberg multiple comparisons.

Analyte	SMA1			SMA2			SMA3		
	T0	T1	T2	T0	T1	T2	T0	T1	T2
	Responder vs Non responder (p value)			*Responder vs Non responder (p value)*			*Responder vs Non responder (p value)*		
Basic FGF	0.476	0.406	0.235	0.445	0.256	0.642	0.468	0.198	0.905
Eotaxin	0.929	0.633	0.265	0.364	0.131	0.248	0.637	0.478	0.239
G-CSF	0.722	0.560	0.125	0.836	0.571	0.424	0.480	0.193	0.059
GM-CSF	0.182	0.672	0.072	0.679	0.924	0.964	0.469	0.906	0.193
IFN-γ	0.689	0.874	0.397	0.483	0.570	0.248	0.814	0.194	0.033
IL-10	0.657	0.958	0.314	0.508	0.296	0.755	0.637	0.236	0.345
IL-12(p70)	0.374	0.874	0.168	0.967	0.251	0.755	0.407	0.474	0.723
IL-13	0.423	0.138	0.186	0.591	0.850	0.965	0.555	0.637	0.034
IL-15	0.859	0.634	0.266	0.563	0.705	0.790	0.480	0.814	0.637
IL-17	0.929	0.874	0.313	0.771	0.394	0.594	0.193	0.547	0.233
IL-1β	1.000	0.788	0.628	0.560	0.154	0.621	0.137	0.198	0.456
IL-1ra	0.722	0.711	0.458	0.869	0.850	0.424	0.408	0.407	0.099
IL-2	0.790	1.000	0.367	0.649	0.394	0.789	0.346	0.631	0.345
IL-4	0.859	0.633	0.596	0.534	1.000	0.858	0.097	0.813	0.813
IL-5	0.859	0.958	0.223	0.772	0.507	0.722	0.239	0.637	0.814
IL-6	0.328	0.266	0.958	0.741	0.450	0.477	0.814	0.478	0.034
IL-7	0.964	0.340	0.749	0.644	0.843	0.323	0.155	0.083	0.904
IL-8	0.155	0.791	0.958	0.509	0.450	0.477	0.814	0.346	0.099
IL-9	0.657	0.874	0.634	0.509	0.257	0.625	0.555	0.637	0.239
IP-10	0.929	0.874	0.125	0.048	0.089	0.248	0.637	0.480	0.099
MCP-1	0.594	0.874	0.368	0.620	0.345	0.424	0.157	1.000	0.034
MIP-1α	0.722	0.791	0.005	0.283	0.450	0.929	0.636	0.813	0.124
MIP-1β	0.722	0.560	0.010	0.457	0.345	0.477	0.814	1.000	0.239
PDGF-BB	0.110	0.223	0.791	0.869	0.014	0.029	0.555	0.813	0.239
RANTES	0.328	0.711	0.711	0.173	0.038	0.328	0.814	0.553	0.814
TNF-α	0.859	0.634	0.427	0.563	0.345	0.449	0.076	0.097	0.906
VEGF	0.859	0.958	0.314	0.741	0.450	0.424	0.480	0.814	0.637
